# Identification of molecular markers associated with the progression and prognosis of endometrial cancer: a bioinformatic study

**DOI:** 10.1186/s12935-020-1140-3

**Published:** 2020-02-19

**Authors:** JinHui Liu, Mingming Feng, SiYue Li, Sipei Nie, Hui Wang, Shan Wu, Jiangnan Qiu, Jie Zhang, WenJun Cheng

**Affiliations:** Department of Gynecology, The First Affiliated Hospital of Nanjing Medical University, 300 Guangzhou Road, Nanjing, 210029 Jiangsu China

**Keywords:** Endometrial cancer, Differentially expressed genes (DEGs), TCGA, GEO, PPI, CMap, Prognosis

## Abstract

**Background:**

Endometrial cancer (EC) is one kind of women cancers. Bioinformatic technology could screen out relative genes which made targeted therapy becoming conventionalized.

**Methods:**

GSE17025 were downloaded from GEO. The genomic data and clinical data were obtained from TCGA. R software and bioconductor packages were used to identify the DEGs. Clusterprofiler was used for functional analysis. STRING was used to assess PPI information and plug-in MCODE to screen hub modules in Cytoscape. The selected genes were coped with functional analysis. CMap could find EC-related drugs that might have potential effect. Univariate and multivariate Cox proportional hazards regression analyses were performed to predict the risk of each patient. Kaplan–Meier curve analysis could compare the survival time. ROC curve analysis was performed to predict value of the genes. Mutation and survival analysis in TCGA database and UALCAN validation were completed. Immunohistochemistry staining from Human Protein Atlas database. GSEA, ROC curve analysis, Oncomine and qRT-PCR were also performed.

**Results:**

Functional analysis showed that the upregulated DEGs were strikingly enriched in chemokine activity, and the down-regulated DEGs in glycosaminoglycan binding. PPI network suggested that NCAPG was the most relevant protein. CMap identified 10 small molecules as possible drugs to treat EC. Cox analysis showed that BCHE, MAL and ASPM were correlated with EC prognosis. TCGA dataset analysis showed significantly mutated BHCE positively related to EC prognosis. MAL and ASPM were further validated in UALCAN. All the results demonstrated that the two genes might promote EC progression. The profile of ASPM was confirmed by the results from immunohistochemistry. ROC curve demonstrated that the mRNA levels of two genes exhibited difference between normal and tumor tissues, indicating their diagnostic efficiency. qRT-PCR results supported the above results. Oncomine results showed that DNA copy number variation of MAL was significantly higher in different EC subtypes than in healthy tissues. GSEA suggested that the two genes played crucial roles in cell cycle.

**Conclusion:**

BCHE, MAL and ASPM are tumor-related genes and can be used as potential biomarkers in EC treatment.

## Background

Endometrial cancer (EC) is the fourth commonest malignancy in females [1]. In 2015, the American Cancer Society (ACS) predicted that the number of new EC cases was 54,870, and 10,170 of them died. This means that in the past 20 years, the mortality of EC has almost doubled. The average age of patients at diagnosis is 63. Among them, 90% are over 50 years old, and only about 20% can get diagnosed before menopause [2]. At present, no useful tool is available to screen EC, hence accurate and early diagnosis is critical for the treatment of EC patients [3–6]. The routine treatment options of EC include surgery, radiotherapy and chemotherapy. Recent years have seen the emergence but not the wide use of EC-related targeted therapy [7]. In recent years, based on the rapid development of high-throughput sequencing technology and increasingly complete public databases, The Cancer Genome Atlas (TCGA) database and Gene Expression Omnibus (GEO) database has collected a large number of clinical, pathological, and biological data from patients with malignancies [8, 9]. Through comprehensive analysis of data, we can more accurately predict the development trend and dig deeper into the mechanism of tumors, providing a reliable research direction for treatment programs [10, 11]. For example, Lin et al. found that a network of RBM8A expression regulated hepatocellular carcinoma [12]. This research screened DEGs using GEO and TCGA data firstly, secondly constructed PPI and co-expression network. Based on cox prognosis analysis, TCGA data, website validation and qRT-PCR, we determined the hub gene and pathways which might be related to EC [13].

## Materials and methods

### Data collection and analysis

**Fig. 1:**
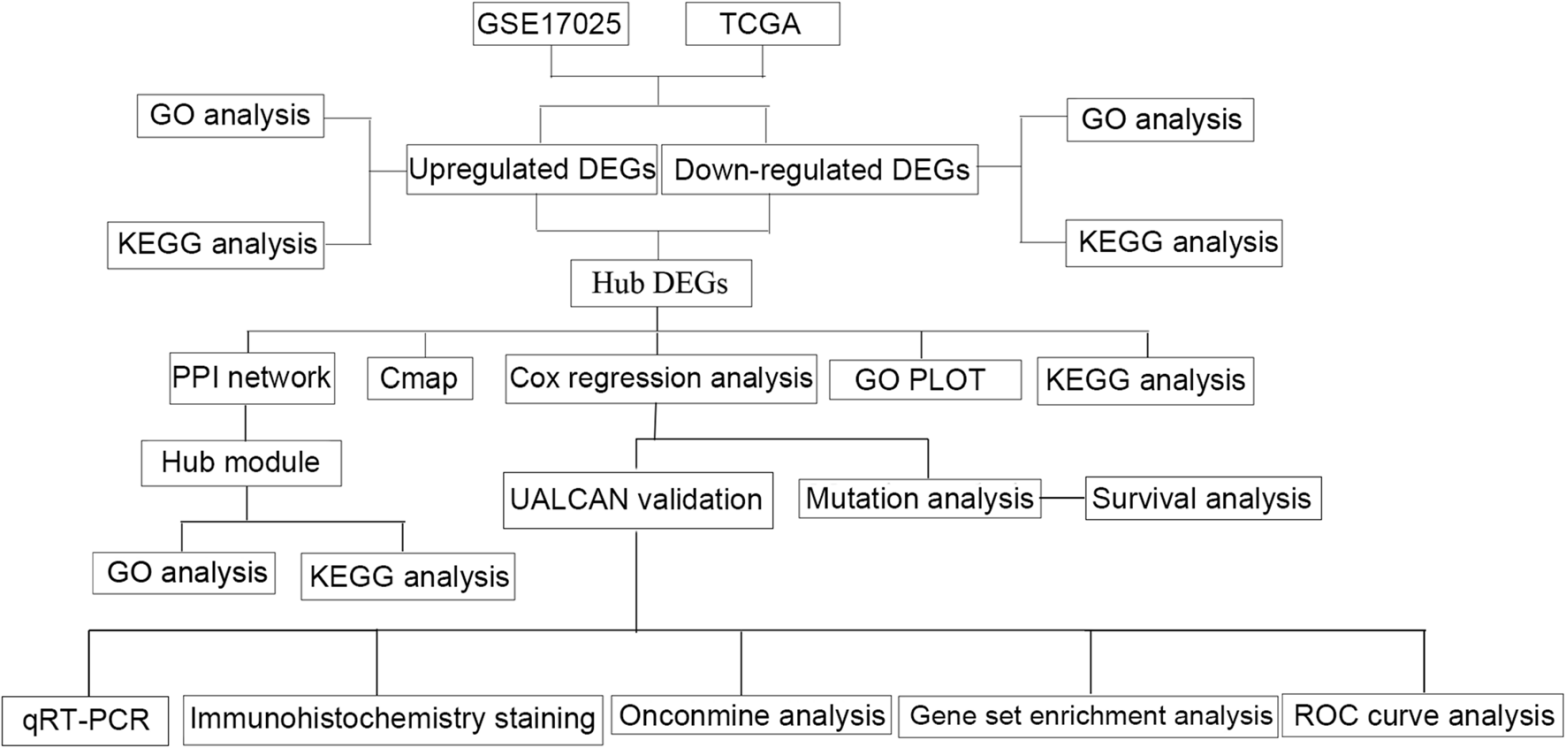
Flow chart of study design

The raw data on GSE17025 and TCGA were integrated. We obtained gene expression profiles form GEO database (http://www.ncbi.nlm.nih.gov/geo/). GSE17025 dataset [14] covered 91 tumor and 12 normal tissue samples. Affymetrix Human Genome U133 Plus 2.0 Array [15] processed raw data. Robust multi-array average (RMA) approach was performed for background correction and normalization [16]. The original GEO data was then converted into expression measures using affy R package [17]. TCGA dataset (https://cancergenome.nih.gov/) was downloaded, covering 35 normal tissue samples and 552 tumor tissues. TCGA (https://cancergenome.nih.gov/) provided the clinical, genomic and mutation data of UCEC and Illumina Hi-Seq RNA-Seq platform provided these RNA sequencing data. These data were downloaded on Feb 01, 2018. The research design was illustrated with a flow chart (Fig. 1).

### Access to DEGs

Package “limma” [18] screened out the DEGs between EC and normal uterus samples. The cut-off criteria was set as The adjusted *p* < 0.05 and |log2fold change (FC)| ≥ 2. For TCGA data, edgeR package was used for DEGs screening [19]. The cut-off criteria was set as FDR < 0.01 and |log2fold change (FC)| ≥ 2. Adjusted *p*-value and FDR were for ruling out false-positives. Online Wayne diagram was for identifying the DEGs simultaneously found in GSE17025 and TCGA. Heatmap was completed by “heatmap” package in R 3.4.4 [20].

### Functional enrichment analysis

DAVID database (https://david.ncifcrf.gov/) is a foundation for high-throughput gene functional analysis. The function and enriched pathways of the proteins encoded by the candidate genes were analyzed and these genes were annotated using DAVID database. GO annotations of the screened DEGs were performed using the DAVID online tool and clusterprofiler [21]. KEGG pathway analysis on DEGs was performed using clusterprofiler. *p*-value < 0.05 was set to be significant.

### Construction and analysis of PPI network complex

STRING (The Retrieval of Interacting Genes Database)(http://www.string-db.org/) provided PPI (protein–protein interaction) information [22]. We used STRING database to explore the interactions between DEGs and visualize the results using Cytoscape software. Cytoscape MCODE plug-in provided access to select hub modules of PPI network [23]. The criteria default parameters as follows: k-core = 2, degree cut-off = 2, max. depth = 100 and node score cut-off = 0.2. For genes in the hub module, we use clusterprofiler again for functional enrichment analysis.

### Identification of potential small molecules

The EC gene signature was queried in CMap. CMap is a computer simulation method for predicting potential drugs that may affect the biological state encoded in gene expression signatures [23]. The DEGs probesets were used to query the CMap database. Finally, the enrichment score indicative of similarity was calculated, ranging from − 1 to 1. A positive connectivity score indicated that a drug could induce the signatured biology in human cell lines. Conversely, a negative connectivity score indicated that a drug could reverse the signatured biology in human cell lines, suggestive of its Possible treatment value. After rank ordering all instances, the connectivity scores were filtered by *p*-value. Tomograph of these relative molecular drugs was researched in Pubchem database (https://pubchem.ncbi.nlm.nih.gov/).

### Construction of a prognostic signature

Univariate Cox proportional hazards regression analyses could provide some Prognosis-related information. With the cutoff of *p* < 0.05, DEGs were seemed to be Prognosis-related. For the top 10 significant prognosis-related genes, construction of multivariate Cox proportional hazards regression model would be helpful. Cox proportional hazards regression with a *p* < 0.05 was set for risk score of developing EC in each patient. According to the mean risk score, patients will be divided into low- and high-risk groups. Kaplan–Meier curve analysis will compare the survival of low-risk and high-risk groups. *p* < 0.05 was the significant cutoff. Receiver operating characteristic (ROC) curve analysis was also performed to estimate the five-year predictive value of the outcomes. The area under the ROC curve was calculated as a predictive value shown as sensitive and specificity.

### Validation of hub genes

R package was for mutation analysis based on the TCGA dataset. The real hub gene was finally validated in UALCAN (http://ualcan.path.uab.edu/analysis.html) [24]. Oncomine 4.5 database (http://www.oncomine.org) was utilized to compare differential expression of common cancer types and their normal adjacent tissues. The HPA (Human Protein Atlas) (http://www.proteinatlas.org/) was used to validate real hub gene [25]. ROC curve analysis was performed in SPSS 23.0 to distinguish normal and cancer tissues.

### Preparation for human EC samples

The study was approved by the Institutional Review Board of Nanjing Medical University. The tissue was removed from the EC patient showing informed consent and immediately stored in an environment of − 80 °C until use. From June 2018 to January 2019, 16 EC tissue samples and 16 normal uterus tissue samples were made in the Department of Gynecology and Obstetrics, the First Affiliated Hospital of Nanjing Medical University including.

### Quantitative real-time RT-PCR (qRT-PCR) analysis

Total RNA was extracted by TRIzol reagent (Invitrogen); RNA 6000 Nano kit and Agilent Bioanalyzer 2100 were used to assess the integrity of the isolated RNA through OD260/280 and OD260/230 ratios, PrimeScript^®^ RT reagent kit was used to react RNA and synthesize single-stranded complementary DNA from RNA according to the manufacturer’s instructions. SYBR^®^ Premix Ex Taq™ Kit (TaKaRa DRR041) was utilized to perform real-time quantification. The cycle threshold (Ct) of each gene was recorded. The relative expression of MAL was calculated as follow: 2^−ΔΔCt^ (ΔCt = Cttarget gene − Ctinternal control). Forward Primer of MAL was “CGCTGCCCTCTTTTACCTCA”. Reverse Primer of MAL was “GAAGCCGTCTTGCATCGTGAT”. GAPDH was used as an endogenous control. Forward primer of GAPDH was “AGAAGGCTGGGGCTCATTTG”. Reverse primer of GAPDH was “AGGGGCCATCCACAGTCTTC”. Quantitative real-time RT-PCR was performed according to the manufacturer’s protocols.

### Gene set enrichment analysis (GSEA)

According to the expression level of hub genes, EC samples from TCGA were divided into 2 different groups. In order to dig out the relative functions, GSEA (http://software.broadinstitute.org/gsea/index.jsp) was used to define the biological processes enriched in the gene rank derived from DEGs between the two groups [26]. Terms with FDR < 0.05 and enriched in all real hub genes were identified.

### Statistical analysis

Two-tailed Student’s t-test was for calculating the difference between subgroups. All analyses were repeated three times. The represented data comes from three separate experiments. Statistical analysis was completed by SPSS 23.0 and R software 3.4.4. *p* < 0.05 was considered statistically significant.

## Results

### Identification of DEGs and the enriched processes

We identified the DEGs in GSE17025 using “limma” with adj. *p* < 0.05 and |log2fold change (FC)| ≥ 2. The top 200 genes in GSE17025 were displayed in the heatmap (Additional file 1: Figure S1). We identified the DEGs in TCGA using the edegr package with FDR < 0.01 and |log2fold change (FC)| ≥ 2. All the genes in TCGA were displayed in the heatmap (Additional file 2: Figure S2). In GSE17025, we screened out 248 DEGs, including 101 up-regulated and 147 down-regulated. All of them were found in EC samples (Additional file 3: Figure S3). In TCGA, we screened out 2614 DEGs from EC samples, which contained 1644 up-regulated and 970 down-regulated. (Additional file 4: Figure S4).

**Fig. 2:**
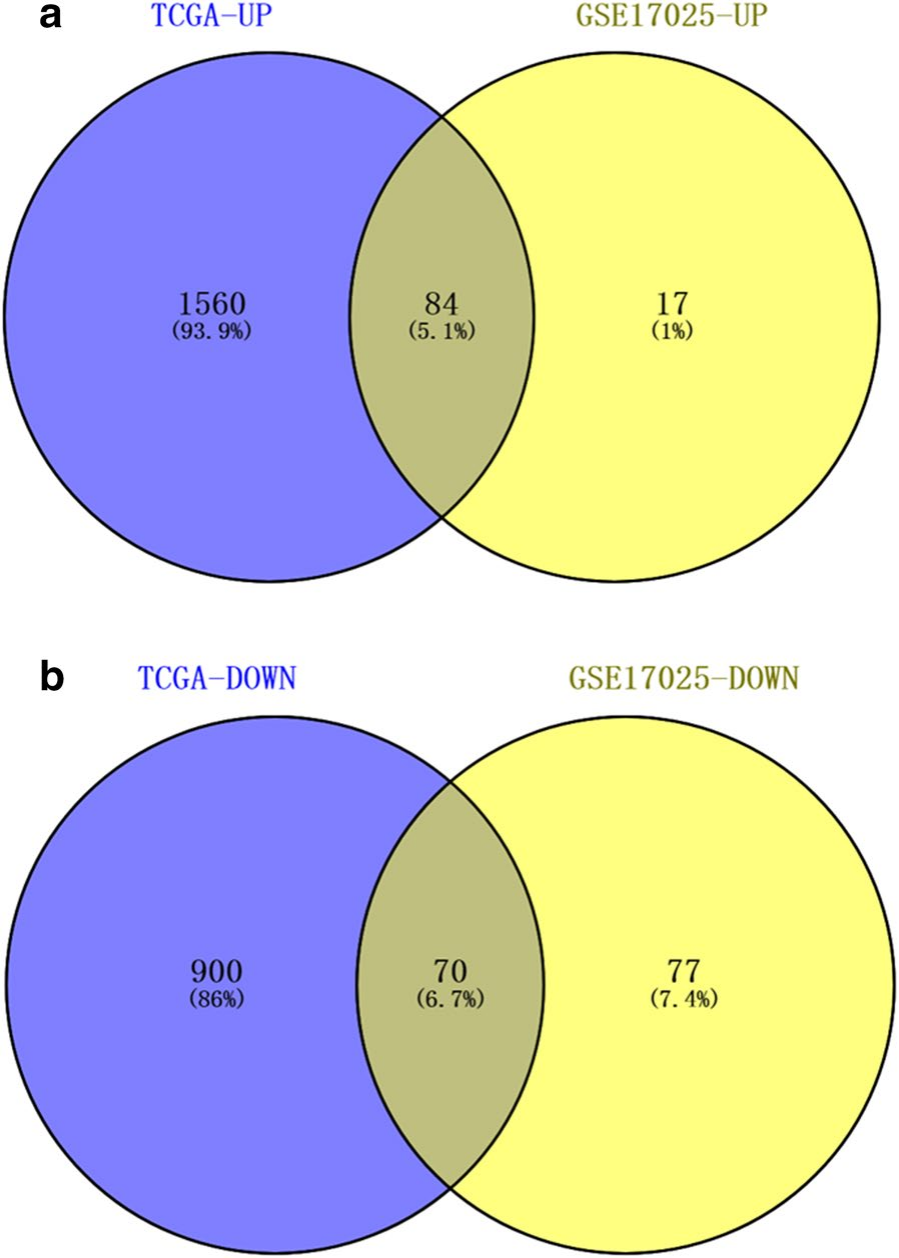
Wayne diagram for comprehensive analysis of GSE17025 and TCGA. **a** Comprehensive analysis of the up-regulated genes in GSE17025 and TCGA, 84 up-regulated hub DEGs. **b** Comprehensive analysis of the down-regulated genes in GSE17025 and TCGA, 70 down-regulated hub DEGs

**Fig. 3:**
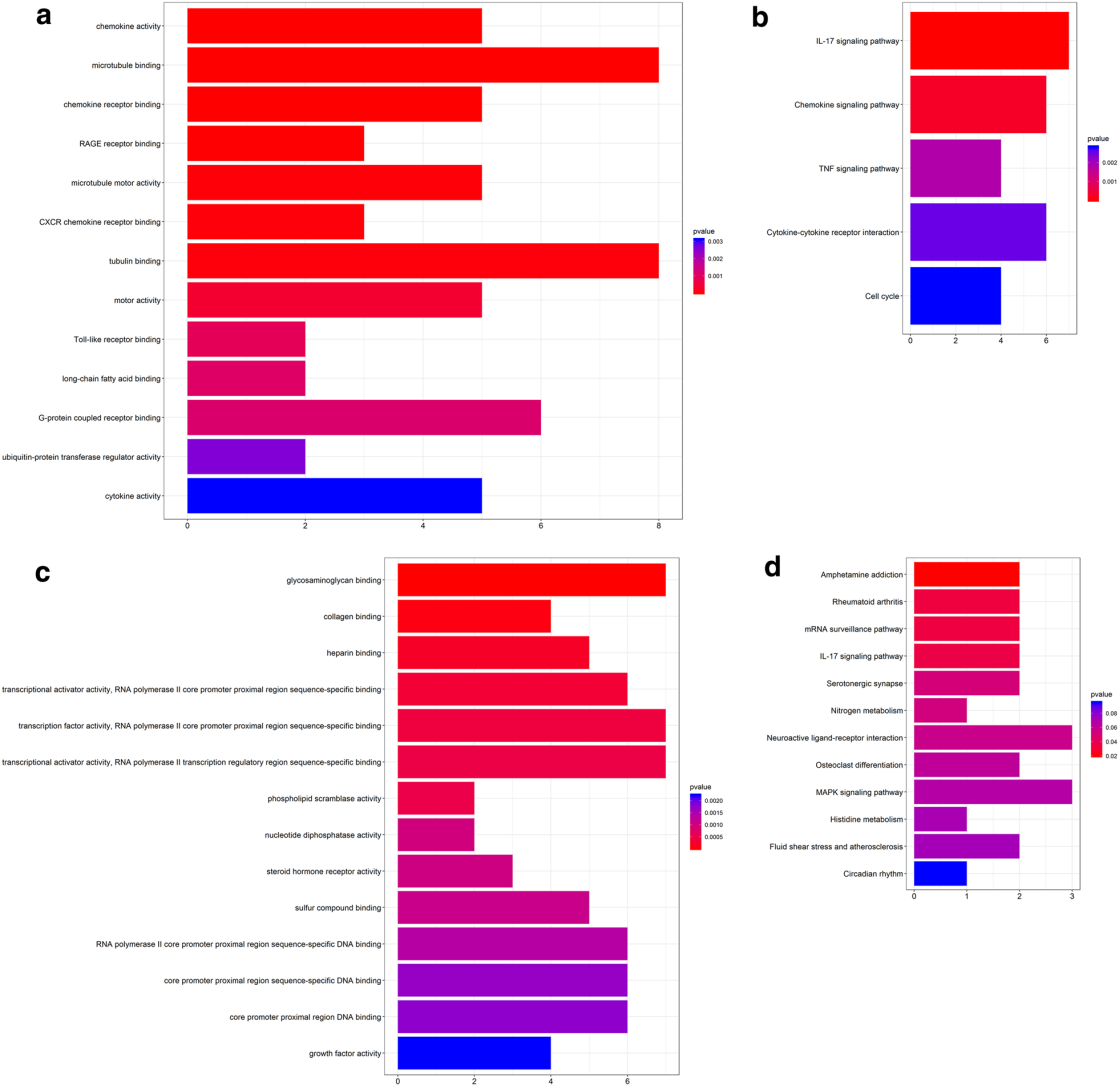
Functional enrichment analysis on the DEGs. **a** GO analysis on the up-regulated DEGs. **b** GO analysis of the down-regulated DEGs. **c** KEGG analysis on the down-regulated DEGs. **d** KEGG analysis on the down-regulated DEGs

Of the DEGs in GSE17025 and TCGA, we screened out 84 up-regulated hub genes (Fig. 2a) and 70 down-regulated hub genes (Fig. 2b). Clusterprofiler was for evaluating the enrichment of gene clusters in biological terms with a cutoff of *p *< 0.05. GO analysis demonstrated that the up-regulated hub genes were mostly enriched in chemokine activity, microtubule binding, chemokine receptor binding, RAGE receptor binding, microtubule motor activity, CXCR chemokine receptor binding and tubulin binding (Fig. 3a); the down-regulated hub genes were highly enriched in glycosaminoglycan binding and collagen binding (Fig. 3b). In KEGG analysis, the up-regulated hub genes were mostly enriched in IL-17 signaling pathway (Fig. 3c); the down-regulated hub genes were highly enriched in Amphetamine addiction (Fig. 3d). We further analyzed these DEGs with adjusted *p *< 0.05 and |logFC| ≥ 2, finding out 154 real DEGs.

### Identification of hub DEGs in EC and functional analysis

**Fig. 4:**
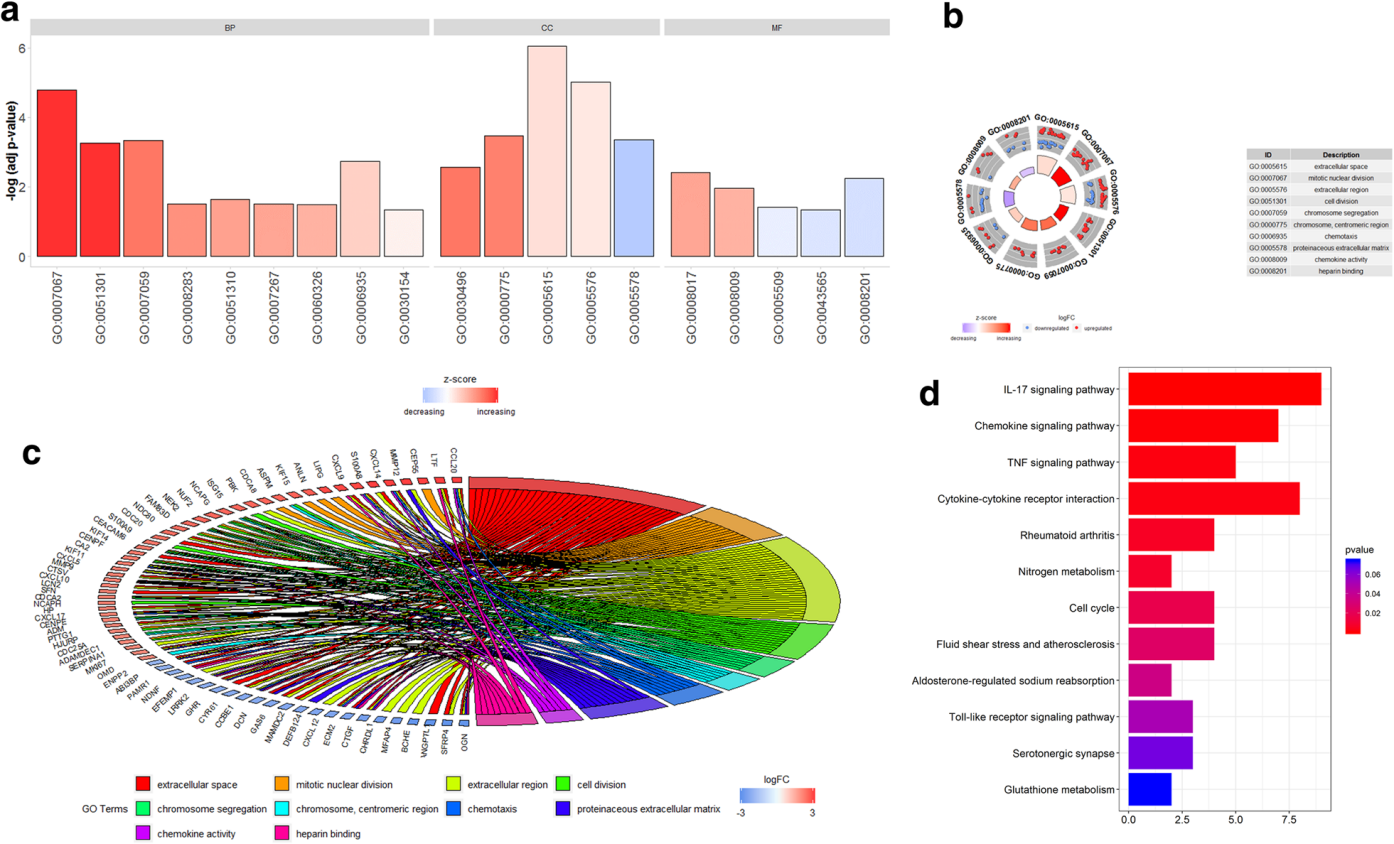
Real hub DEGs associated biological processes and KEGG pathways. **a**–**c** GO PLOT of the real hub DEGs revealed terms pertaining to cancer and cellular functions. **d** KEGG analysis on the real hub DEGs

To clarify the functions of these DEGs, we first explored the associated biological processes and KEGG pathways in TCGA and GEO datasets. The most enriched GO terms pertaining to biological process (BP), cellular, component (CC) and molecular function (MF) are shown in Fig. 4a. The most enriched GO term in BP was “mitotic nuclear division” (*p* < 0.05), that in CC was “extracellular space” (p < 0.05), and that in MF was “microtubule binding” (*p* < 0.05) (Fig. 4b). We further obtained 10 significantly enriched GO terms with a *p*-value < 0.05 (Fig. 4c).

KEGG analysis showed that the DEGs were strikingly enriched in IL-17 signaling pathway, Chemokine signaling pathway and TNF signaling pathway (Fig. 4d).

### PPI network and analysis on clusters

**Fig. 5:**
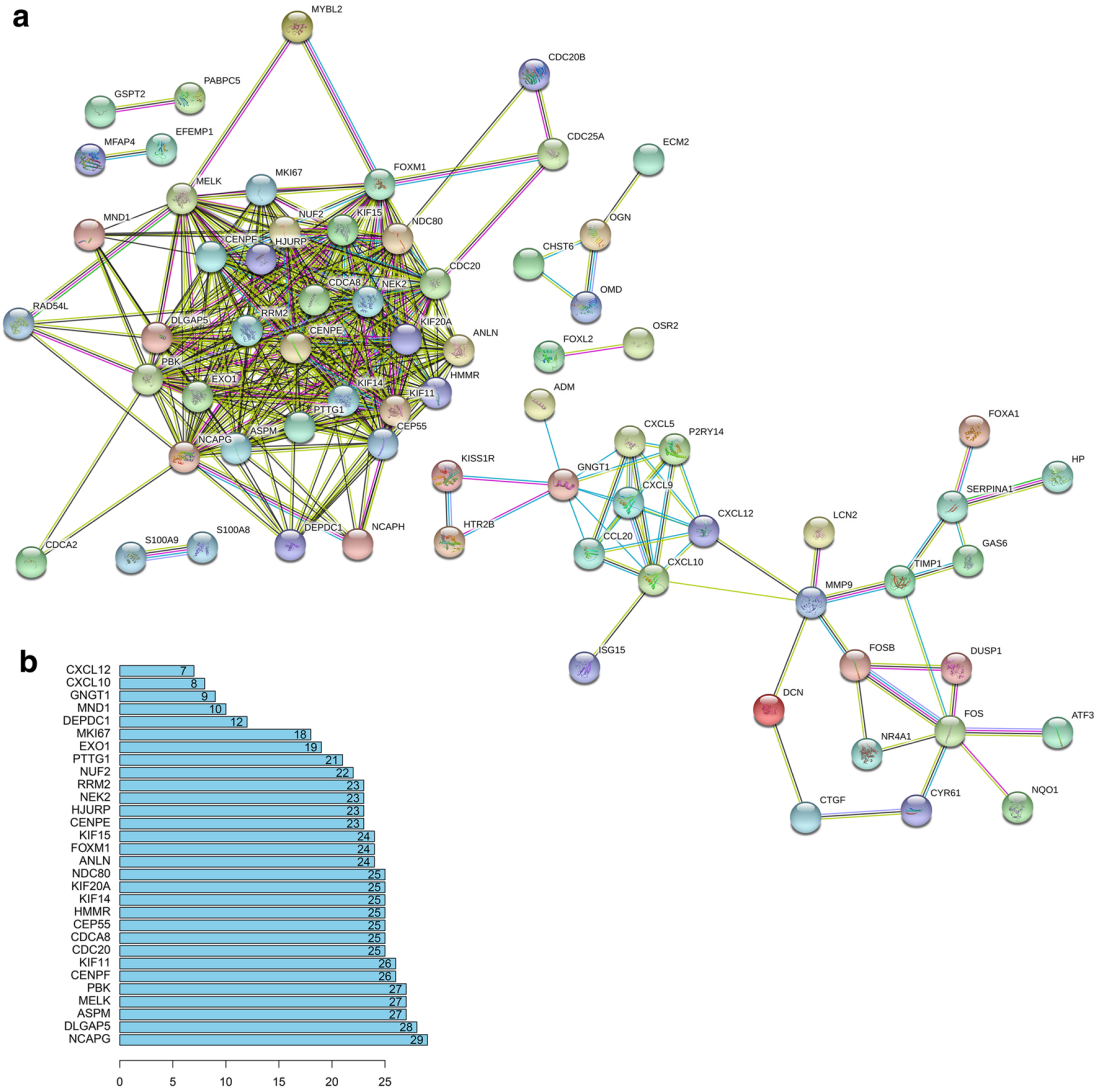
Cluster analysis of the PPI network. **a** 154 DEGs were filtered into the DEGs PPI network complex that contained 154 nodes and 382 edges. **b** Histogram of key proteins. The y-axis represents the name of genes, the x-axis represent the number of adjacent genes, and height is the number of gene connections

STRING mapped 154 DEGs into PPI network containing 154 nodes and 382 edges (Fig. 5a). A total of 30 prominent proteins were identified, with estrogen NCAPG being the most important protein contacting 29 nodes (Fig. 5b).

**Fig. 6:**
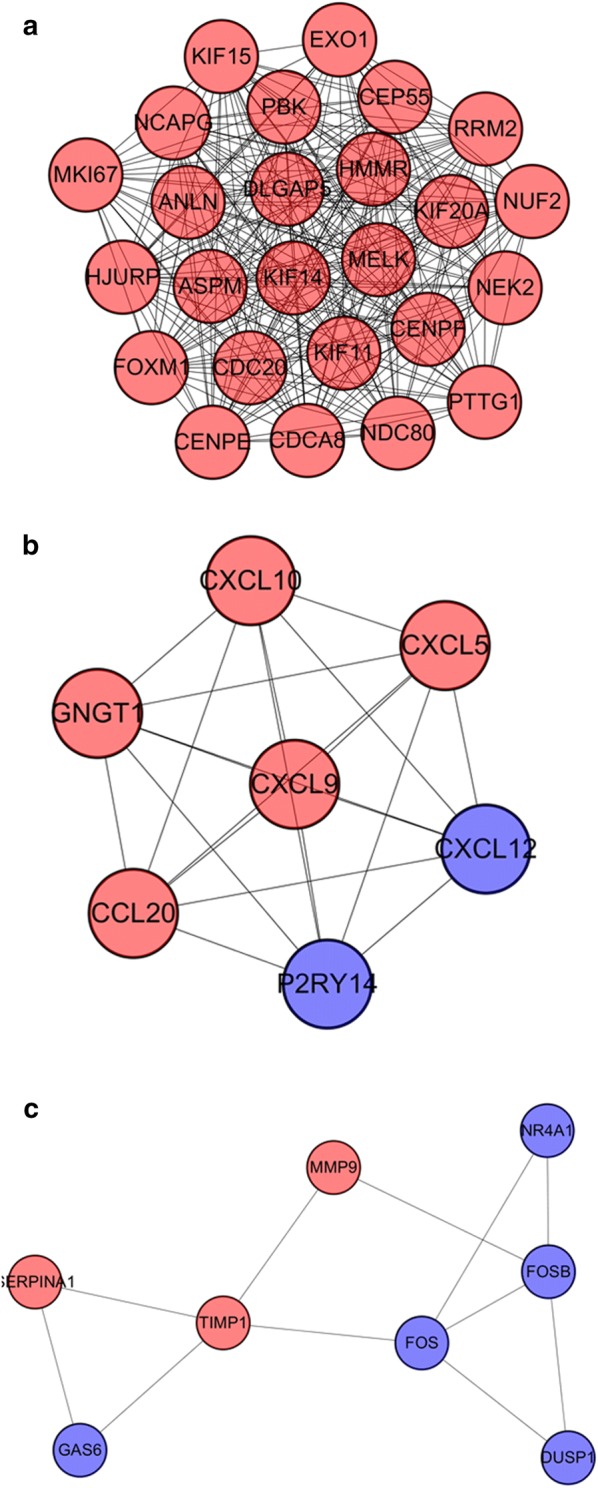
Module analysis of PPI network. The red node represents the up-regulated gene and the blue node represents the down-regulated gene. **a** Module rank 1. This cluster consists of 25 nodes and 284 edges and has the highest score. **b** Module rank 2. This cluster consists of 7 nodes and 21 edges and has the second highest score. **c** Module rank 3. This cluster consists of 8 nodes and 11 edges and has the third highest score

Then MCODE was used to find clusters in the network. Four clusters were calculated according to k-core = 2. Among them, cluster 1 contained 25 nodes and 284 edges, with the highest score (Fig. 6a), cluster 2 contained 7 nodes and 21 edges (Fig. 6b), cluster 3 contained 8 nodes and 11 edges (Fig. 6c), cluster 4 contained 3 nodes and 3 edges. These results suggested that the 154 DEGs had effects on EC.

**Fig. 7:**
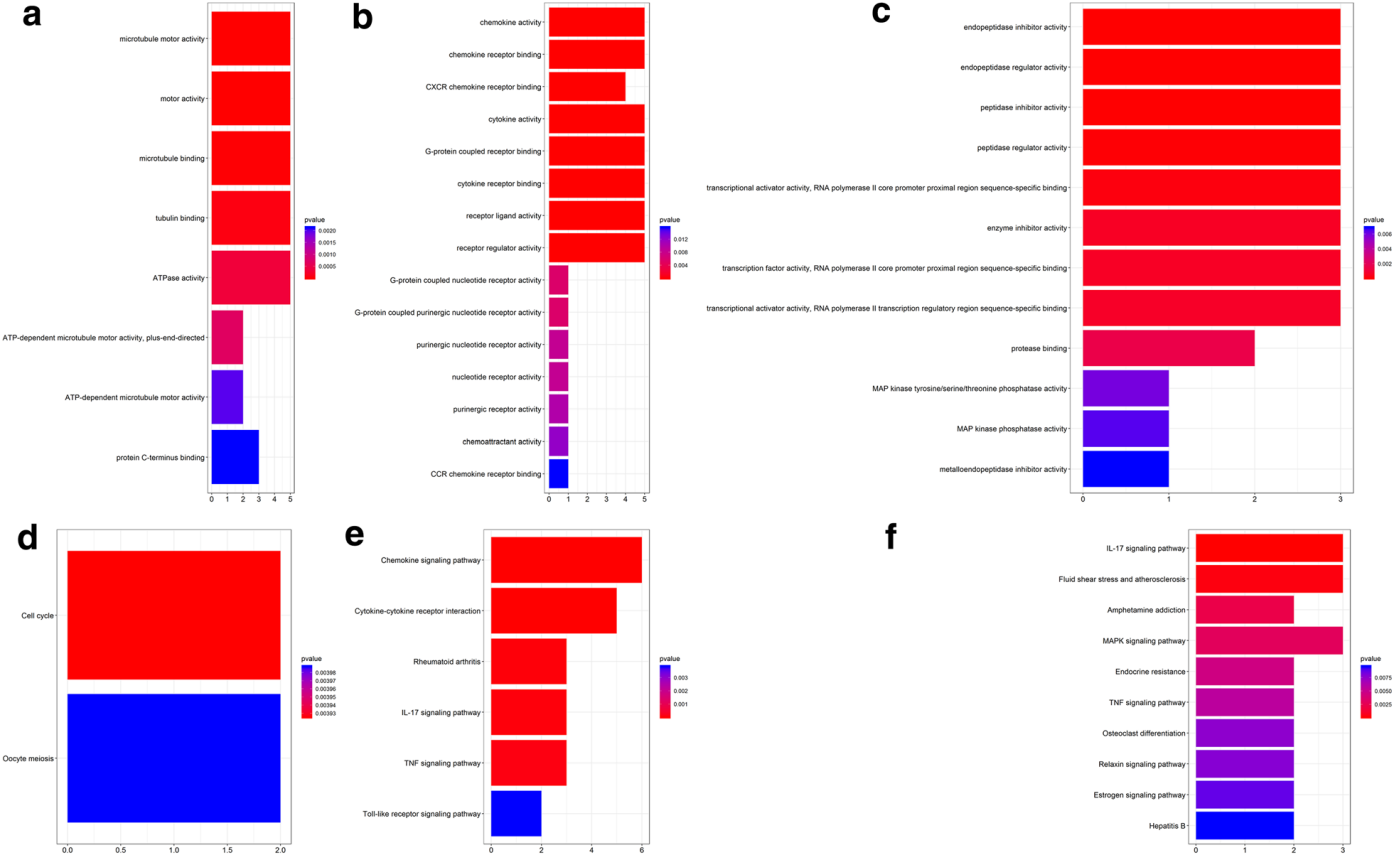
GO and KEGG analyses on the hub modules. **a** GO analysis on module 1. **b** GO analysis on module 2. **c** GO analysis of module 3. **d** KEGG analysis on module 1. **e** KEGG analysis on module 2. **f** KEGG analysis on module 3

We performed the functional analysis for the top 3 clusters. In GO analysis, the DEGs of cluster 1 were mostly enriched in microtubule motor activity, motor activity and microtubule binding (Fig. 7a); the DEGs of cluster 2 in chemokine activity, chemokine receptor binding, CXCR chemokine receptor binding, cytokine activity, G-protein coupled receptor binding, cytokine receptor binding and receptor ligand activity (Fig. 7b); the DEGs of cluster 3 in endopeptidase inhibitor activity, endopeptidase regulator activity, peptidase inhibitor activity, peptidase regulator activity and transcriptional activator activity, RNA polymerase II core promoter proximal region sequence-specific binding (Fig. 7c). KEGG analysis showed that the DEGs of cluster 1 were mostly enriched in cell cycle (Fig. 7d); the DEGs of cluster 2 in chemokine signaling pathway and cytokine–cytokine receptor interaction (Fig. 7e); the DEGs of cluster 3 in IL-17 signaling pathway (Fig. 7f).

### Potential small molecule drugs

**Fig. 8:**
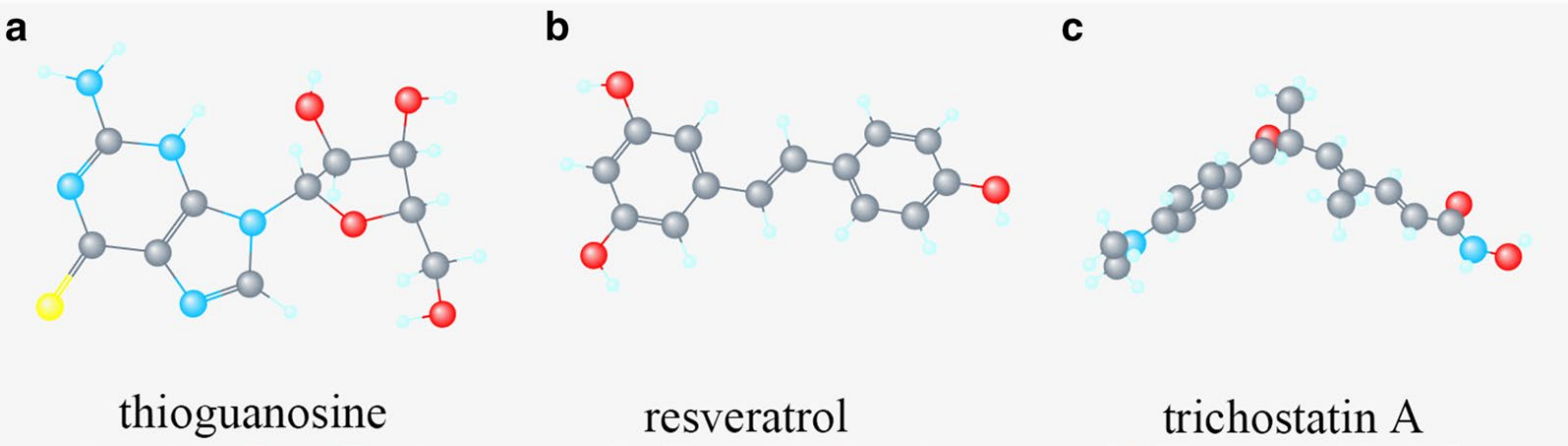
Top 3 molecule drugs. **a** thioguanosine, **b** resveratrol, **c** trichostatin A

CMap compared EC samples with healthy controls to screen out small molecule drugs. Strong negative correlation was found between EC and thioguanosine, resveratrol, trichostatin A, 0175029-0000, trifluoperazine and LY-294002; strong positive correlation was found between EC and viomycin, adiphenine, clorsulon and heptaminol (Additional file 5: Figure S5). These drugs might have therapeutic effects on EC. The tomographes of the top 3 associated molecule drugs were investigated in Pubchem database (Fig. 8a–c).

### Identification of prognostic signature

**Fig. 9:**
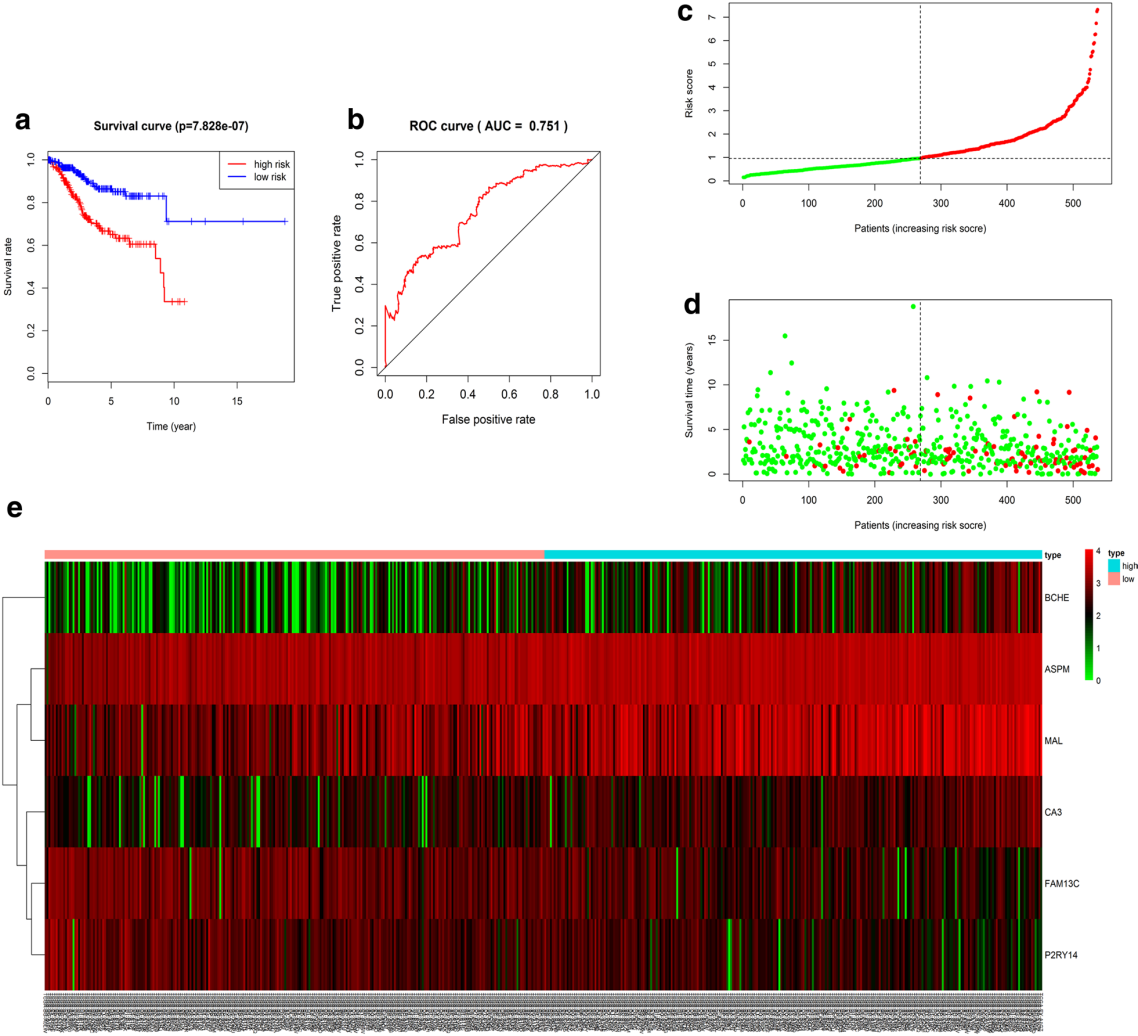
Survival prognosis model on the 6 hub genes. **a** Survival analysis showed that the patients in the high risk group had worse overall survival than those in low risk group in TCGA cohort. **b** ROC analysis was performed to calculate the most optimal cutoff value to divide the EC patients into high risk and low risk group. **c**, **d** The risk scores for all patients in TCGA cohort are plotted in ascending order and marked as low risk (blue) or high risk (red), as divided by the threshold (vertical black line). **e** Six expression and risk score distribution in TCGA cohort by z-score, with red indicating higher expression and light blue indicating lower expression

Of the 154 DEGs, the univariate Cox proportional hazards regression analysis screened out the top 10 EC-relative genes, including MAL, BCHE, P2RY14, ASPM, CA3, FAM13C, FAM83D, CXorf57, SFN and CPED1 (Additional file 6: Figure S6); multivariate Cox proportional hazards regression analysis was further performed on the 10 genes, which screened BCHE, FAM13C, CA3, P2RY14, ASPM and MAL (Additional file 7: Figure S7). The risk score for predicting overall survival was calculated as follows: Risk score = 0.126 * BCHE − 0.121 * FAM13C + 0.136 * CA3 − 0.139 * P2RY14 + 0.231 * ASPM + 0.0892 * MAL. According to the risk score, patients were divided into low- and high-risk groups. Survival analysis showed that low-risk patients had longer overall survival than high-risk patients in TCGA cohort (Fig. 9a). The AUC of five-year survival ROC curve analysis was 0.751 (Fig. 9b). The distribution of risk score, survival status, and the expression of six genes of each patient were also analyzed (Fig. 9c–e). The expression level of the six genes in low- and high-risk groups was shown in Additional file 8: Figure S8. Meanwhile, we analyzed the relationship between the different clinical parameters and the risk score based on six genes. The univariate and multivariate Cox proportional hazards regression showed that only tumor status together with the risk score based on six genes was independent prognostic indictor of EC (Table 1). The heatmap showed the expression levels of the six genes in high- and low-risk groups based on the TCGA dataset. We observed significant between-group differences in tumor status, grade, histological type, age and stage (*p* < 0.001) (Additional file 9: Figure S9).

**Table 1:** Univariate analysis and multivariate analysis of the correlation of six-gene expression with OS among endometrial cancer patients

Parameter	Univariate analysis			Multivariate analysis		
	HR	95% CI	p value	HR	95% CI	p value
Age (≤ 60 vs > 60)	1.788	1.118–2.859	*0.015*	1.566	0.956–2.566	0.075
Stage (stage I & stage II vs stage III & stage IV)	4.070	2.70–6.205	*0.000*	1.604	0.993–2.591	0.053
Histological_type (endometrioid vs mix & serous)	2.997	1.972–4.553	*0.000*	0.840	0.489–1.442	0.527
Grade (G1 & G2 vs G3 & G4)	3.395	1.975–5.835	*0.000*	1.544	0.834–2.857	0.167
Tumor_status (with tumor vs tumor free)	11.042	7.05–17.300	*0.000*	7.795	4.682–12.978	*0.000*
RiskScore	1.543	1.369–1.739	*0.000*	1.195	1.022–1.398	*0.026*

Italic values indicate *p* < 0.05

*HR* hazard ratio, *CI* confidence interval

### Hub gene validation

**Fig. 10:**
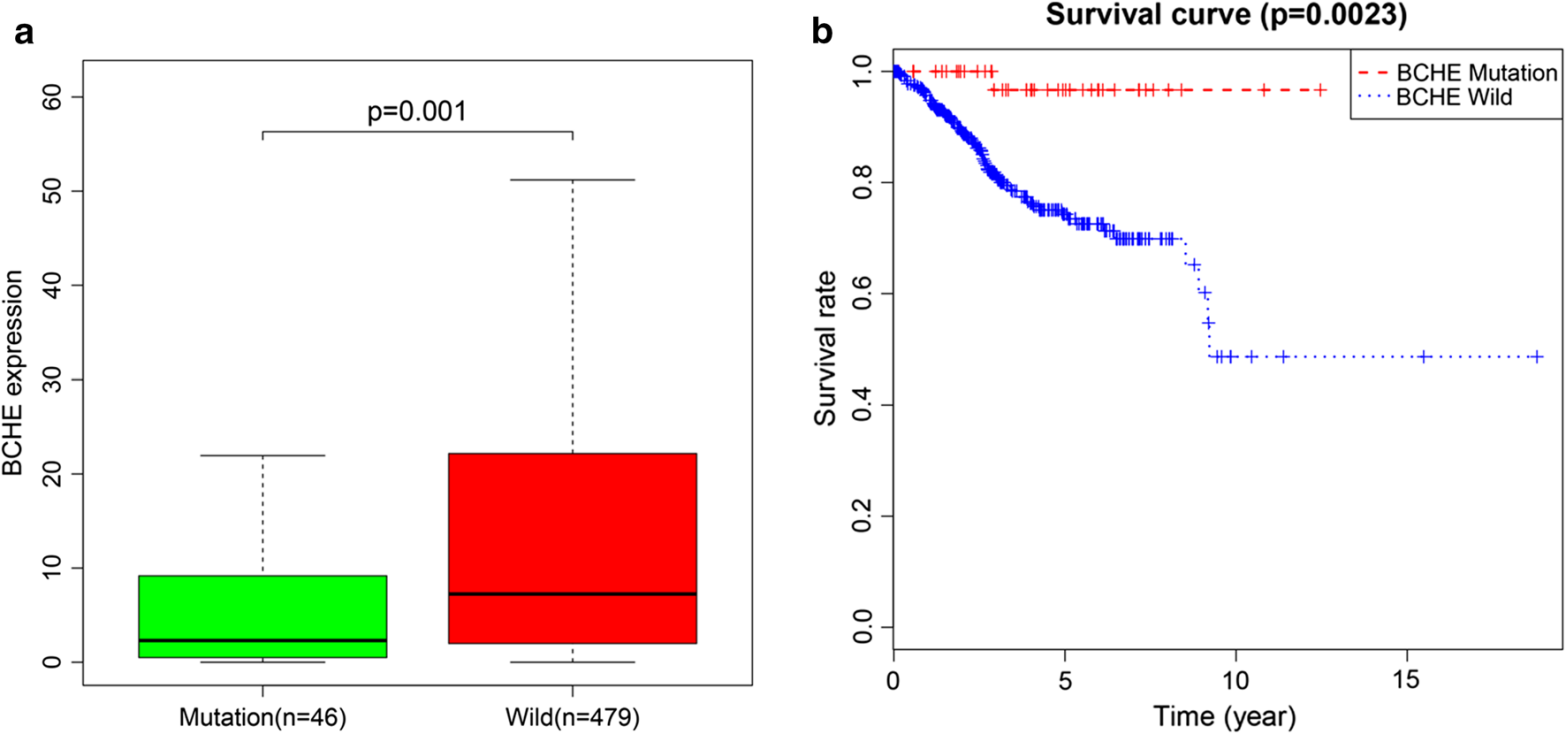
Validation of BCHE. **a** Mutation analysis of BCHE. **b** Mutation of BCHE was positively related to EC overall survival

Based on TCGA dataset and using R language, we performed mutation analysis on BCHE, ASPM and MAL which exhibited significant prognostic value (*p *< 0.05). We found that BCHE showed significant mutation (Fig. 10a). We further found that patients with BCHE mutation had a better prognosis (Fig. 10b), suggesting that BCHE mutation may be a protective factor for EC patients.

**Fig. 11:**
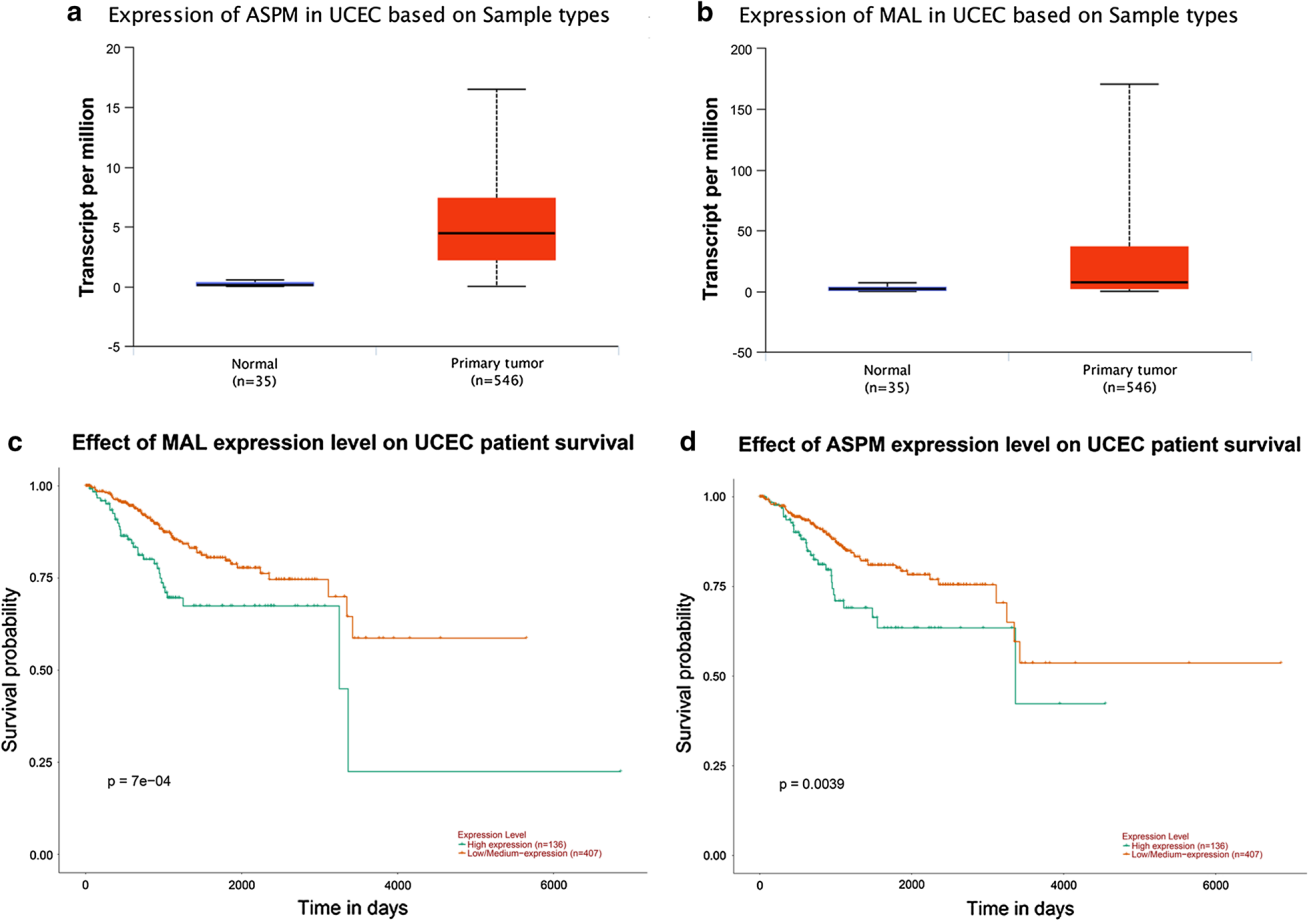
Validation of UALCAN website. **a**, **b** The expression of MAL and ASPM in EC tissues of primary tumor are all higher than normal tissues. **c**, **d** Survival analysis of MAL and ASPM

**Fig. 12:**
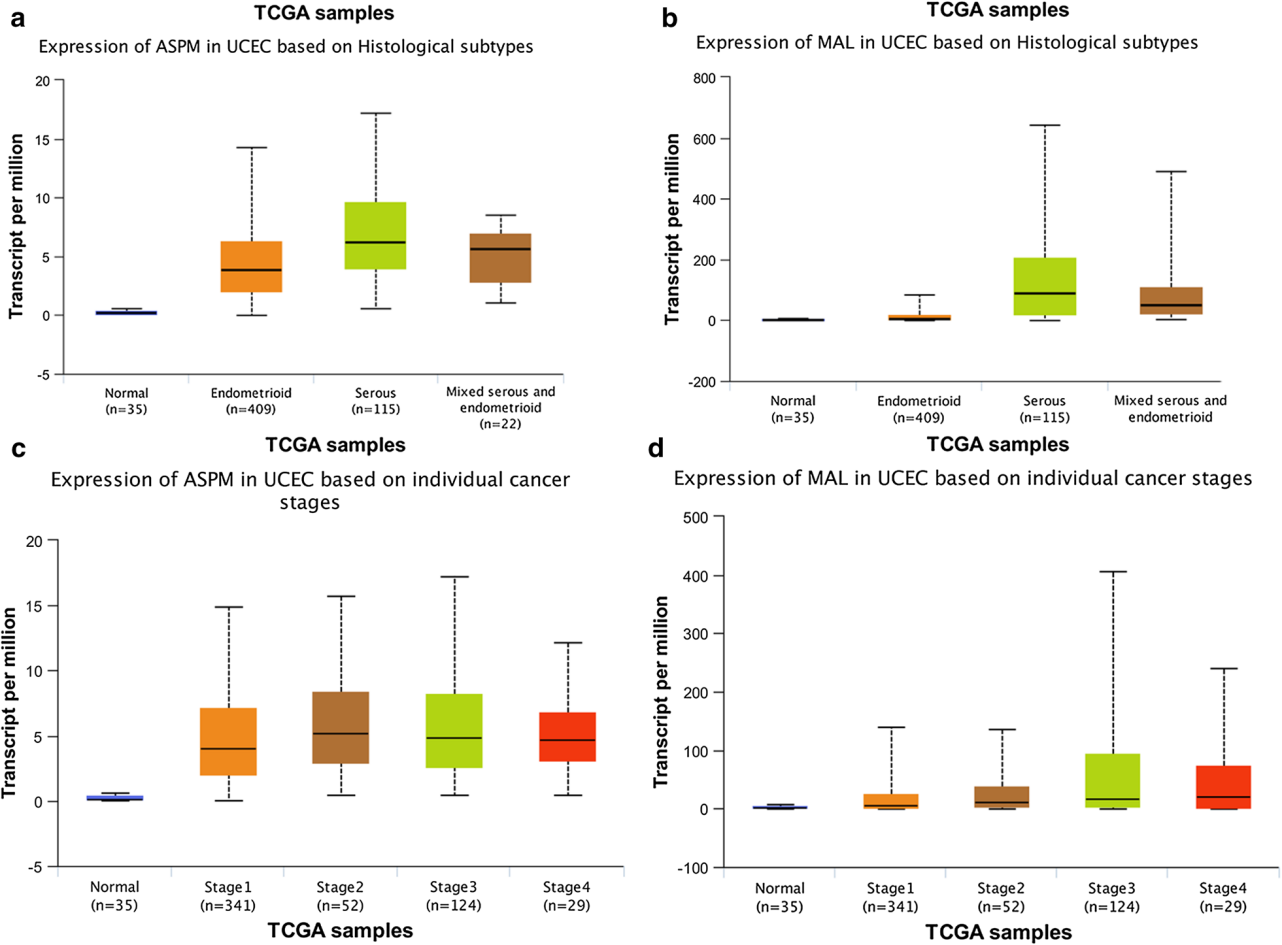
Validation of UALCAN website. **a**, **b** The expression of MAL and ASPM in EC tissues of different histological subtypes are all higher than normal tissues. **c**, **d** The expression of MAL and ASPM in EC tissues at different stages are all higher than normal tissues

**Fig. 13:**
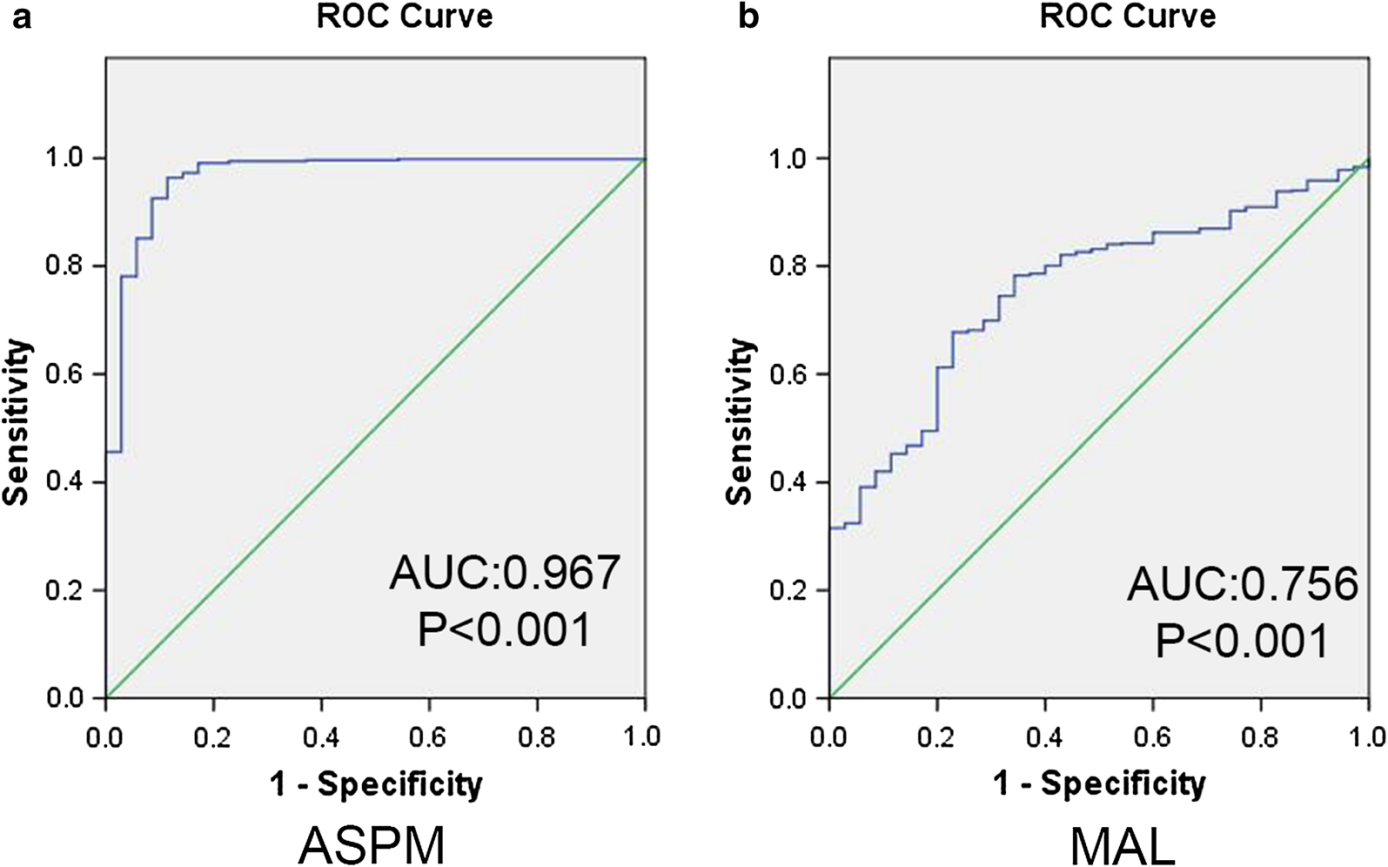
ROC curve analysis and AUC statistics were implemented to evaluate the capacity of MAL and ASPM to distinguish EC from normal tissues. **a** ASPM. **b** MAL

**Fig. 14:**
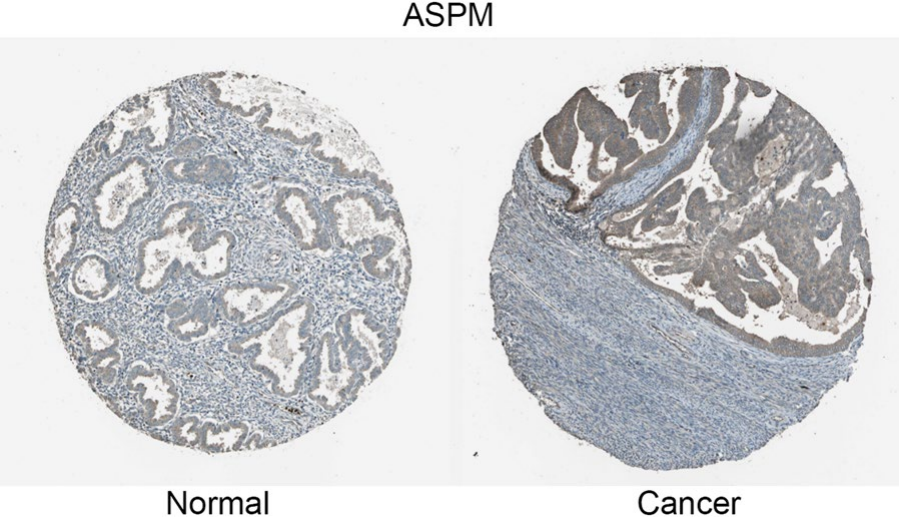
Immunohistochemistry of ASPM based on the Human Protein Atlas. Protein levels of ASPM in normal tissue (staining: Low; intensity: Weak; quantity: 75–25%; Location: Cytoplasmic/membrano). Protein levels of ASPM in tumor tissue (staining: Medium; intensity: Moderate; quantity: > 75%; Location: Cytoplasmic/membrano)

**Fig. 15:**
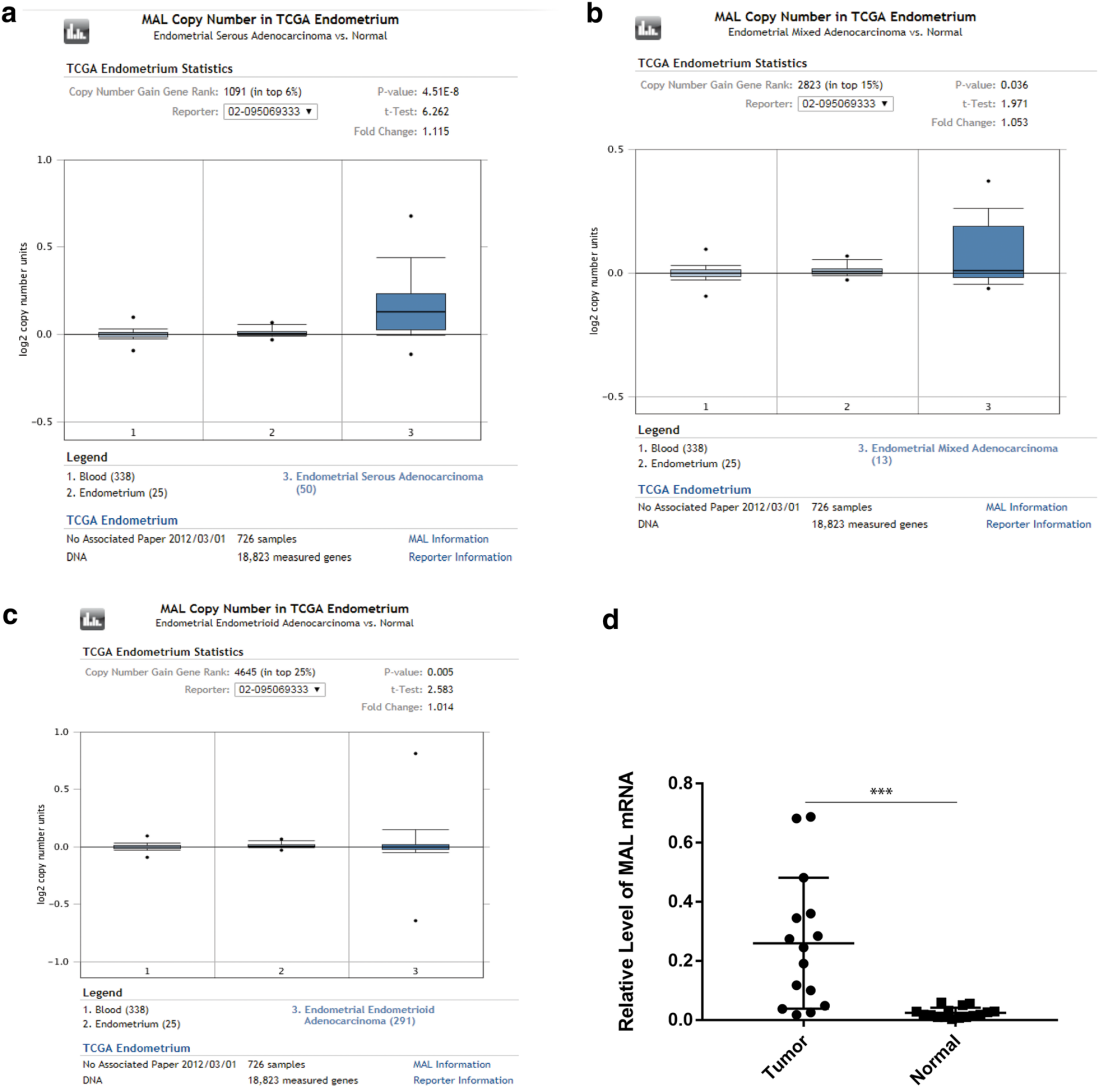
MAL transcription in EC (Oncomine) and the validation in clinical samples. **a** Box plot showing MAL copy number in The Cancer Genome Atlas (TCGA) Endometrium and Endometrial serous Adenocarcinoma dataset. **b** Box plot showing MAL copy number in The Cancer Genome Atlas (TCGA) Endometrium and Endometrial mixed Adenocarcinoma dataset. **c** Box plot showing MAL copy number in The Cancer Genome Atlas (TCGA) Endometrium and Endometrial endometrioid Adenocarcinoma dataset. **d** The relative expression level of MAL in clinical samples using qRT-PCR

Using UALCAN, we found that MAL and ASPM expressed higher in tumor than in normal tissues (Fig. 11a, b), both negatively related to the overall survival of the EC patients (Fig. 11c, d). In addition, both had higher expression levels in EC tissues of different subtypes, such as serous carcinoma, endometrioid adenocarcinoma and mixed serous and endometrioid adenocarcinoma (Fig. 12a, b). Their expression levels also increased at different stages of EC (Fig. 12c, d). Finally, ROC curve analysis was for evaluating the capacity of MAL and ASPM, so as to distinguish EC from normal tissues (Fig. 13). MAL was missing in the immunohistochemistry database. Immunohistochemistry staining showed the higher expression of ASPM in the tumor sample compared with the normal sample (Fig. 14). Data in the Oncomine 4.5 database revealed that DNA copy number variation (CNV) of MAL was significantly higher in different subtypes of EC tissues than in normal tissues (p ˂ 0.01). Although the fold change of DNA CNV was within 2, MAL ranked within the top 5% (Fig. 15a–c). We further validate the expression of MAL in clinical tissues using qRT-PCR. Interestingly, the relative expression level of MAL was significantly elevated in tumor tissue than in normal tissue (Fig. 15d).

### Gene set enrichment analysis (GSEA)

**Fig. 16:**
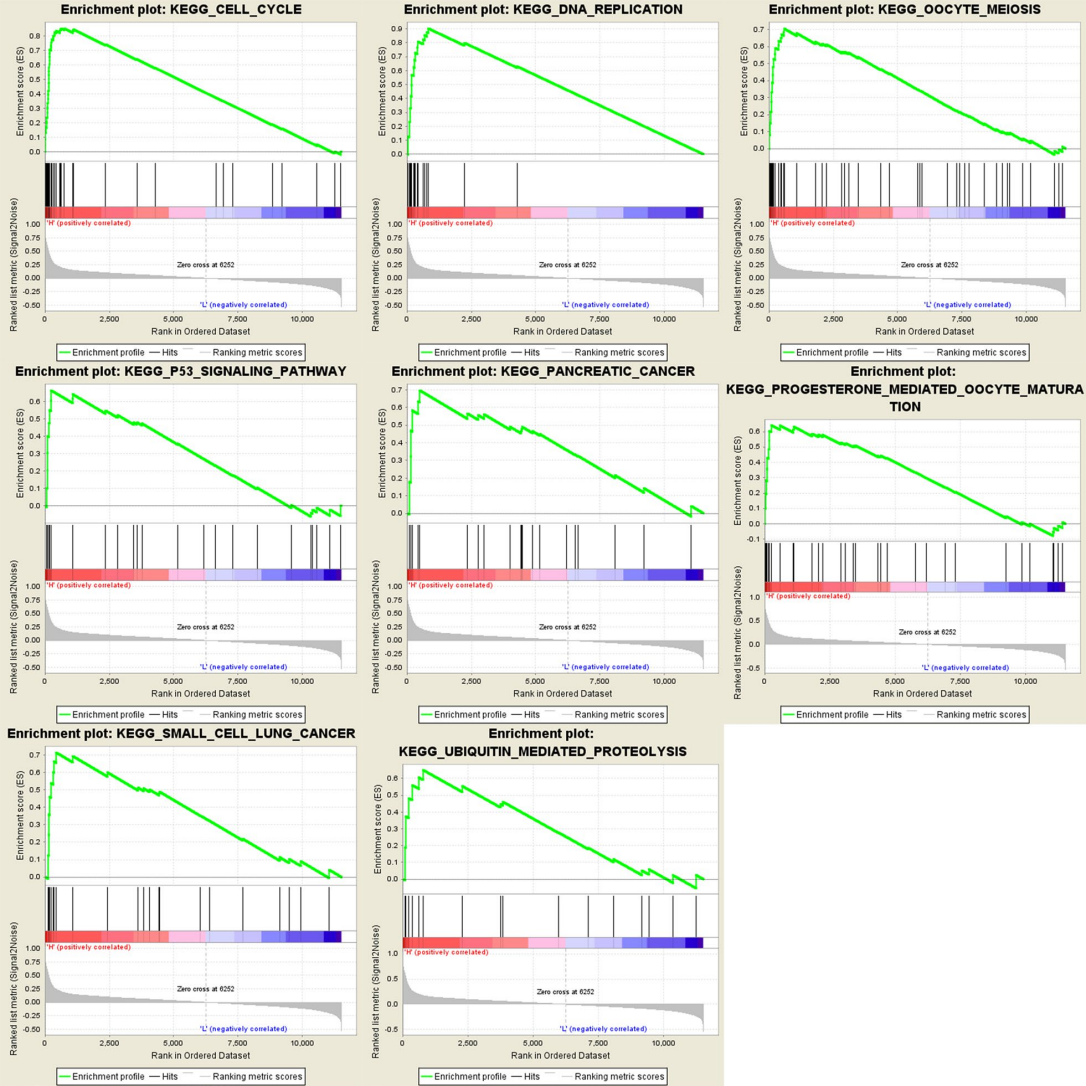
GSEA using TCGA UCEC databases. The eight most functional gene sets enriched in EC samples with ASPM highly expressed

**Fig. 17:**
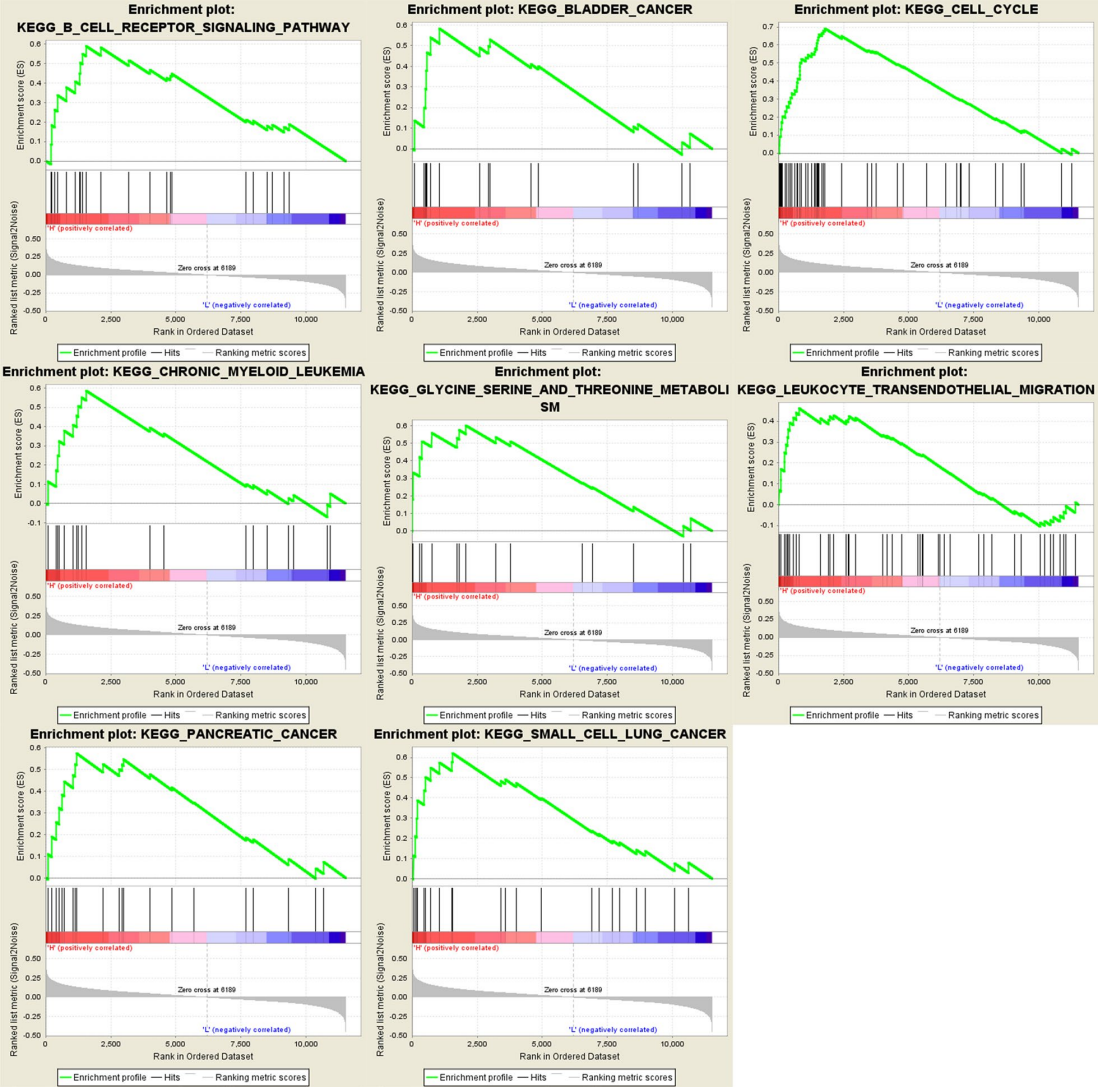
GSEA using TCGA UCEC databases. The eight most functional gene sets enriched in EC samples with MAL highly expressed

To identify the potential function of MAL and ASPM in EC, GSEA was conducted to search the enriched KEGG pathways. For ASPM, “cell cycle”, “DNA replication”, “oocyte meiosis”, “p53 signaling pathway”, “pancreatic cancer”, “progesterone mediated oocyte maturation”, “small cell lung cancer”, “ubiquitin mediated proteolysis” were enriched in eight gene sets (n = 552) (Fig. 16). For MAL, “B cell receptor signaling pathway”, “bladder cancer”, “cell cycle”, “chronic myeloid leukemia”, “glycine serine and threonine metabolism”, “leukocyte transendothelial migration”, “pancreatic cancer”, “small cell lung cancer” were enriched in eight gene sets (n = 552) (Fig. 17) (FDR < 0.05).

## Discussion

In this study, we screened out EC-related DEGs based on GSE17025 and TCGA datasets. The up-regulated DEGs were strikingly enriched in chemokine activity and IL-17 signaling pathway, and the down-regulated DEGs in glycosaminoglycan binding and Amphetamine addiction. We further screened out 154 hub DEGs, most of which were enriched in mitotic nuclear division, extracellular space, microtubule binding and IL-17 signaling pathway.

Liu et al. have found the chemokine activity in hepatocellular carcinoma metastasis [27]. IL-17 signaling pathway acts in the progression of lung cancer and liver cancer [28, 29]. Microtubule binding is involved in the development of colorectal cancer [30]. All these findings point out the possible correlation between some DEGs and EC development, which may provide a new direction for EC research.

We also found NCAPG was a functional protein in EC. NCAPG has shown its regulatory property in digestive tract tumors [31–33]. Some small EC-countering molecules have been identified. Among them, thioguanosine, resveratrol and trichostatin A have shown tight association with EC development. Thioguanosine can regulate the basal activity of leukemia cells [34] in. Resveratrol was proved to affect ovarian, breast and digestive tract tumors [35–38]. Trichostatin A, as a histone deacetylase (HDAC) inhibitor, exhibits anticancer effects when used in combination with radiotherapy or chemotherapy [39, 40].

We found that ASPM, BCHE and MAL were highly EC-prognosis-related. BCHE mutation showed positive correlation with EC prognosis. Using UALCAN and TCGA datasets, we found the higher expression of MAL and ASPM in EC tissue than in normal tissue, and their expression levels were negatively related to the overall survival of the EC patients. In addition, MAL and ASPM had higher expression levels in EC tissues of different subtypes, such as serous carcinoma, endometrioid adenocarcinoma and mixed serous and endometrioid adenocarcinoma. Their expression levels also increased with EC stage. Finally, ROC curve analysis was conducted for evaluating the capacity of MAL and ASPM in distinguishing EC from normal tissues. Immunohistochemistry staining demonstrated the higher expression of ASPM in EC samples, compared with that in the normal samples. Data in the Oncomine 4.5 database revealed that DNA copy number variation (CNV) of MAL was significantly higher in different subtypes of EC tissues than in the healthy tissues. We further validated the expression of MAL in clinical tissues using qRT-PCR. Interestingly, the relative expression level of MAL was significantly elevated in tumor tissues compared to the normal tissues. GSEA enrichment analysis showed that MAL and ASPM were mostly associated with cell cycle.

Butyrylcholinesterase (BChE) is a plasma enzyme that hydrolyzes ghrelin and bioactive esters. Its modulation in prostate cancer development has been proven [41]. Bernardi et al. found that amplification and deletion of BCHE was related to cholinesterase genes in sporadic breast cancer [42].

A study has confirmed that MAL serves as a bridge between TLR2/TLR4- and MyD88-mediated signaling to orchestrate downstream inflammatory responses and regulate intestinal homeostasis and colitis-associated colorectal cancer in mice [43]. Choi et al. found that MAL was significantly down-regulated and methylated in gastric cancer tissues [44]. Van Baars et al. found that MAL methylation in cervical scrapes could indicate the status of underlying lesion. Zanotti et al. found that in high-grade serous ovarian carcinoma, MAL overexpression predicted chemoresistance and poor prognosis [45, 46]. In the present study, MAL as the hub gene also showed its aberrant expression in EC development, a finding that can guide the future exploration into EC mechanism.

In prostate and hepatocellular cancers, ASPM promoted the progression and exacerbated the prognosis, which means ASPM might be a novel marker for cancer progression [47, 48]. Researches showed that ASPM worsened the prognosis of ovarian cancer in many ways [49, 50]. ASPM can also increase the aggressiveness of pancreatic tumor [51]. In the present study, we verified the prognostic value of ASPN in EC, which broadens the landscape of ASPM research.

There are some highlights of our study. First of all, we obtained hub genes by taking the intersection between DEGs of TCGA and GEO,finding that BCHE, MAL and ASPM are tumor-related genes and can be used as potential biomarkers in EC treatment. Second, the prognostic model in our study can effectively predict EC patients’ outcomes, which provide a new method to evaluate patients’ prognosis. Third, Thioguanosine, resveratrol and trichostatin A can be used as antagonists against EC.

However, there are some limitations in this study. For example, our research was actually an analysis based on previous data; therefore, additional experimental studies in vivo and vitro are needed. In the future, based on the results of this study, we will design PCR, Western blotting and immunohistochemistry tests to explore the molecular mechanisms. Second, the clinical sample size of PCR was not large enough. Finally, the therapeutic effects of candidate drugs targeting DEGs should be verified. In all, more well-designed studies should be carried out to support our findings.

## Conclusion

In conclusion, BCHE, MAL and ASPM may be potential prognostic markers in EC. Thioguanosine, resveratrol and trichostatin A can be used as antagonists against EC.

## Supplementary information

**Additional file 1: Figure S1.** Heatmap of top 200 genes in GSE17025.

**Additional file 2: Figure S2.** Heatmap of all genes in TCGA.

**Additional file 3: Figure S3.** The volcano plot of all DEGs in GSE17025. Red dots represent up-regulated genes, green dots represent down-regulated genes, and black dots represent genes without differential expression.

**Additional file 4: Figure S4.** The volcano plot of all DEGs in TCGA. Red dots represent up-regulated genes, green dots represent down-regulated genes, and black dots represent genes without differential expression.

**Additional file 5: Figure S5.** Results of CMap analysis.

**Additional file 6: Figure S6.** Univariate Cox proportional hazards regression analysis showed the top 10 EC-relative genes.

**Additional file 7: Figure S7.** Multivariate Cox proportional hazards regression analysis further screened out 6 hub genes.

**Additional file 8: Figure S8.** Expression of the six genes in low- and high-risk groups based on TCGA dataset .Red represents high-risk groups, blue represents low-risk groups.

**Additional file 9: Figure S9.** The heatmap of the six-gene expression levels between high- and low-risk groups in clinical information based on the TCGA dataset.

## Data Availability

Not applicable.

## Abbreviation

EC: Endometrial cancer
GEO: Gene expression omnibus
DEGs: Differentially expressed genes
STRING: Search Tool for the Retrieval of Interacting Genes Database
PPI: Protein–protein interaction
MCODE: Molecular Complex Detection
ROC: Receiver operating characteristic
GSEA: Gene set enrichment analysis
RMA: Robust multi-array average
HR: Hazard ratio
CI: Confidence interval
