# Supplementary figures and images for: Identification of molecular markers associated with the progression and prognosis of endometrial cancer: a bioinformatic study
Source: Cancer Cell Int. 2020 Feb 19;20:59. doi: 10.1186/s12935-020-1140-3 (PMC7031962; doi:10.1186/s12935-020-1140-3)

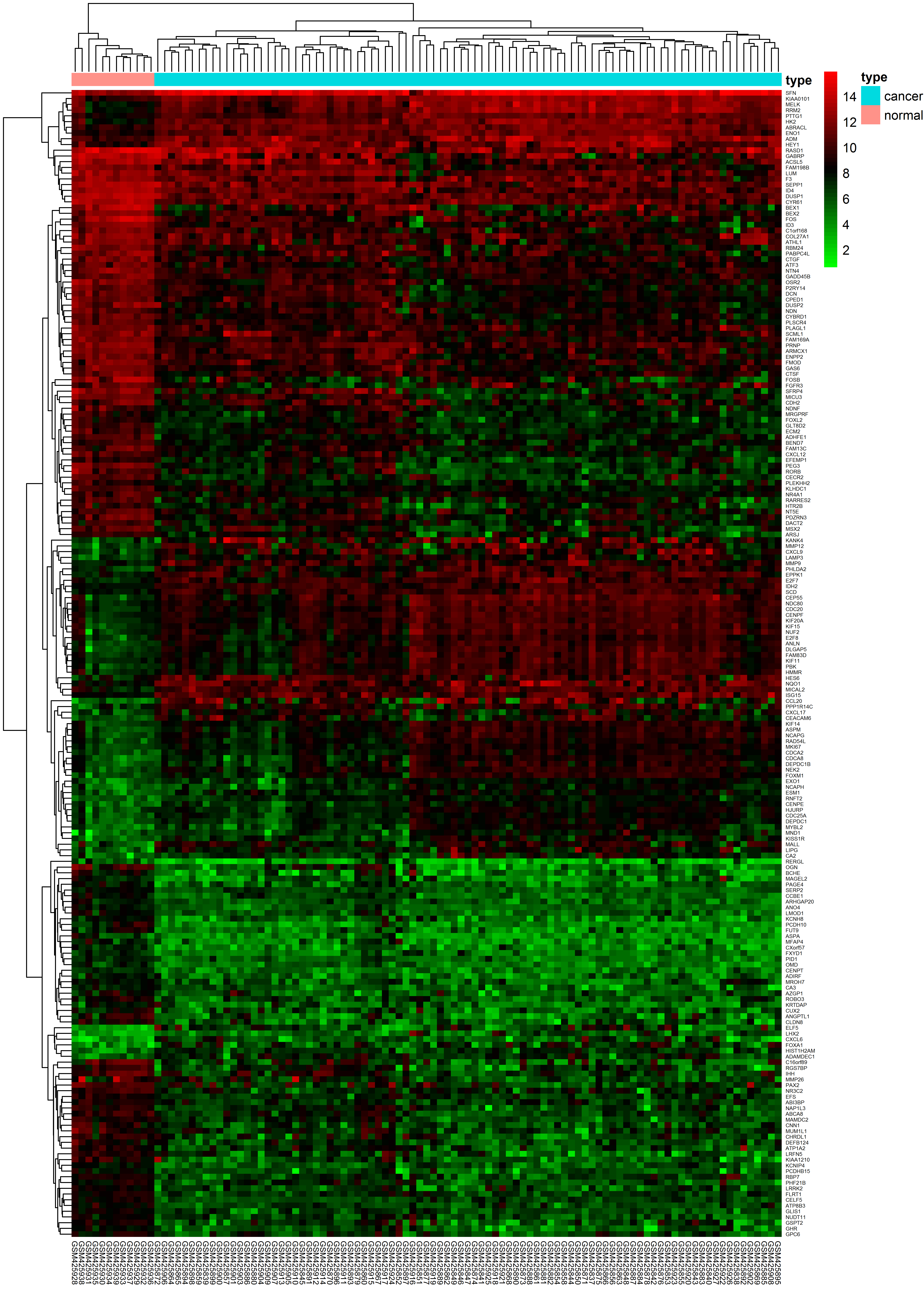

Supplement: Supplementary file 1 — Additional file 1: Figure S1. Heatmap of top 200 genes in GSE17025. [file 12935_2020_1140_MOESM1_ESM.tiff]

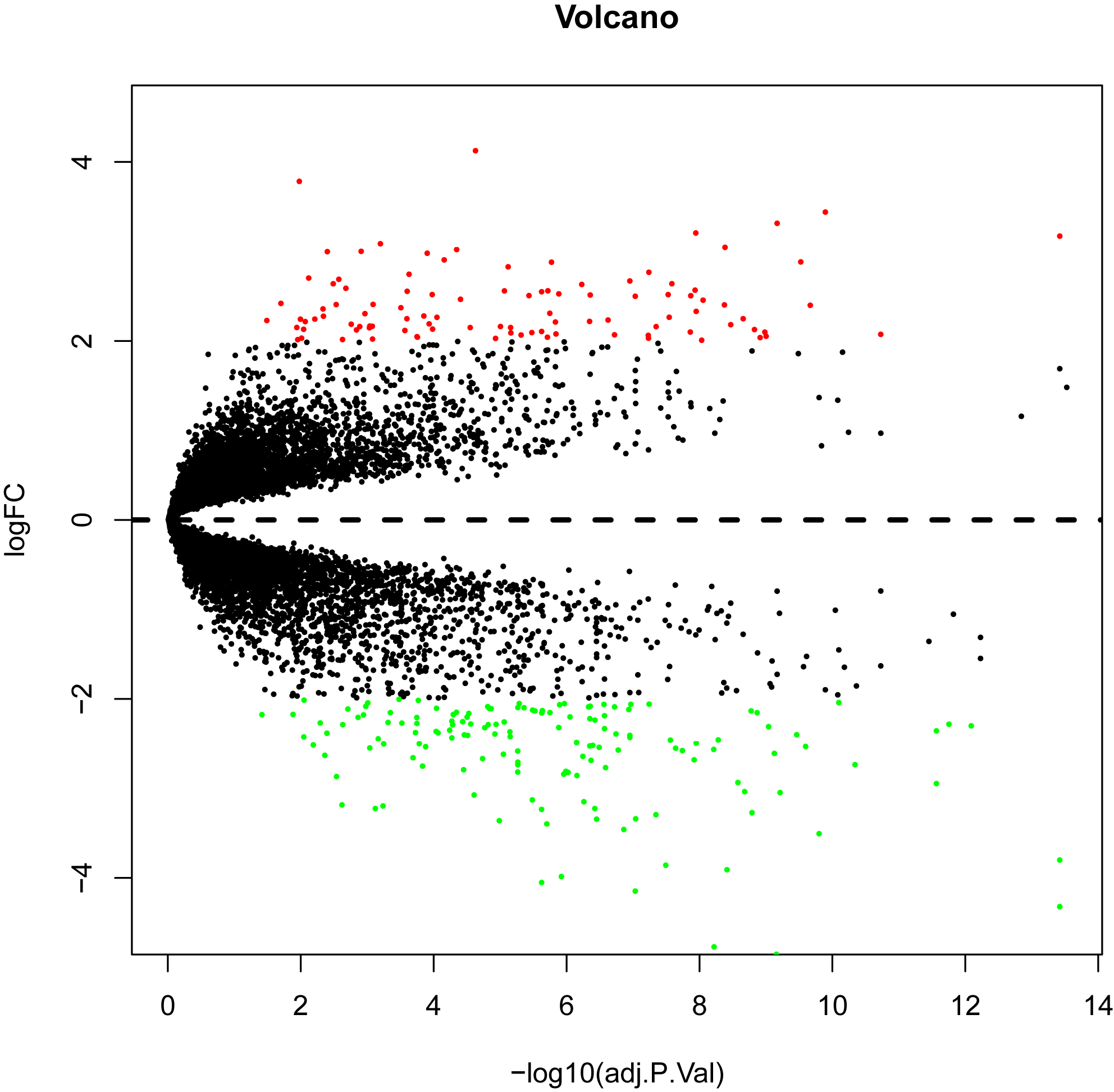

Supplement: Supplementary file 3 — Additional file 3: Figure S3. The volcano plot of all DEGs in GSE17025. Red dots represent up-regulated genes, green dots represent down-regulated genes, and black dots represent genes without differential expression. [file 12935_2020_1140_MOESM3_ESM.tif]

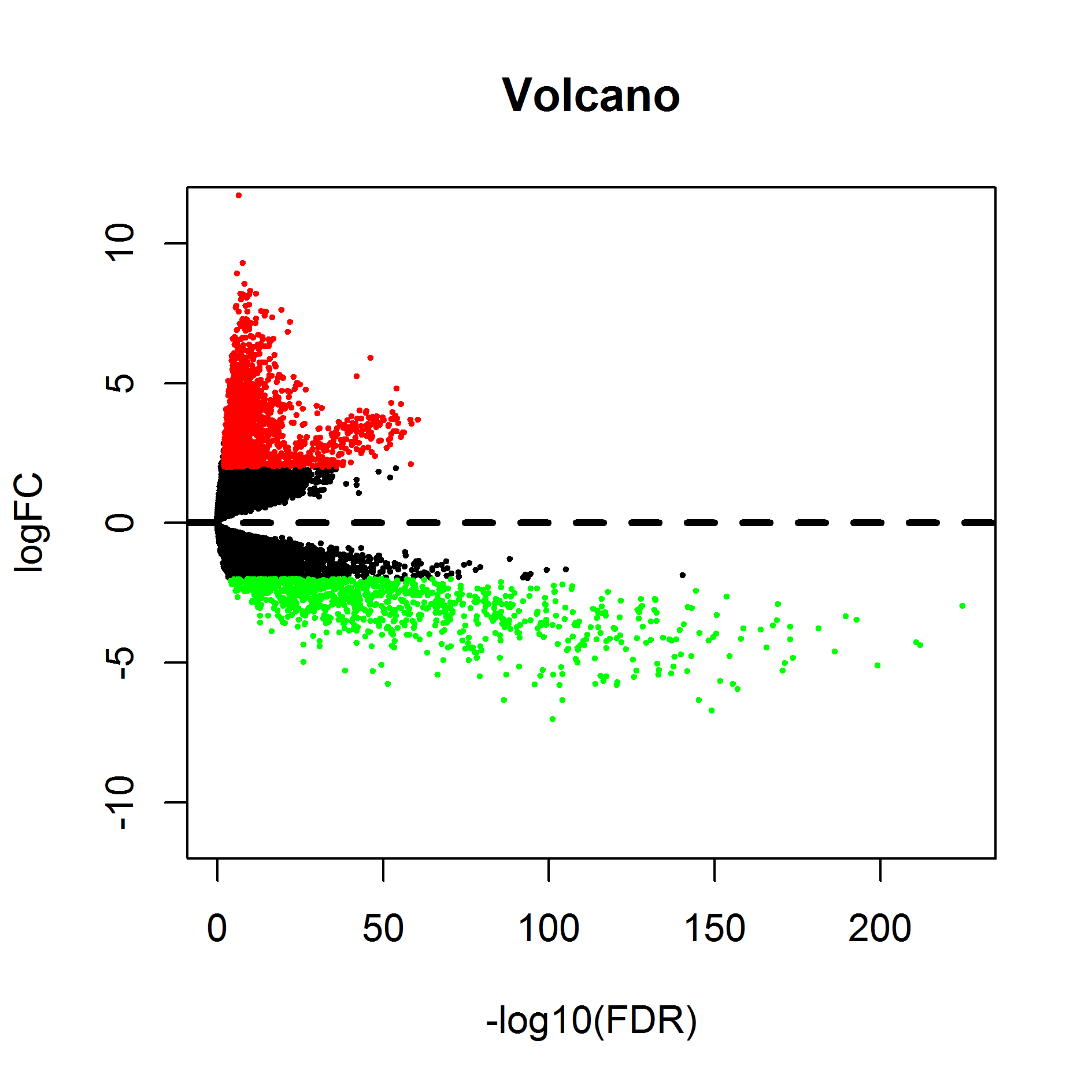

Supplement: Supplementary file 4 — Additional file 4: Figure S4. The volcano plot of all DEGs in TCGA. Red dots represent up-regulated genes, green dots represent down-regulated genes, and black dots represent genes without differential expression. [file 12935_2020_1140_MOESM4_ESM.tiff]

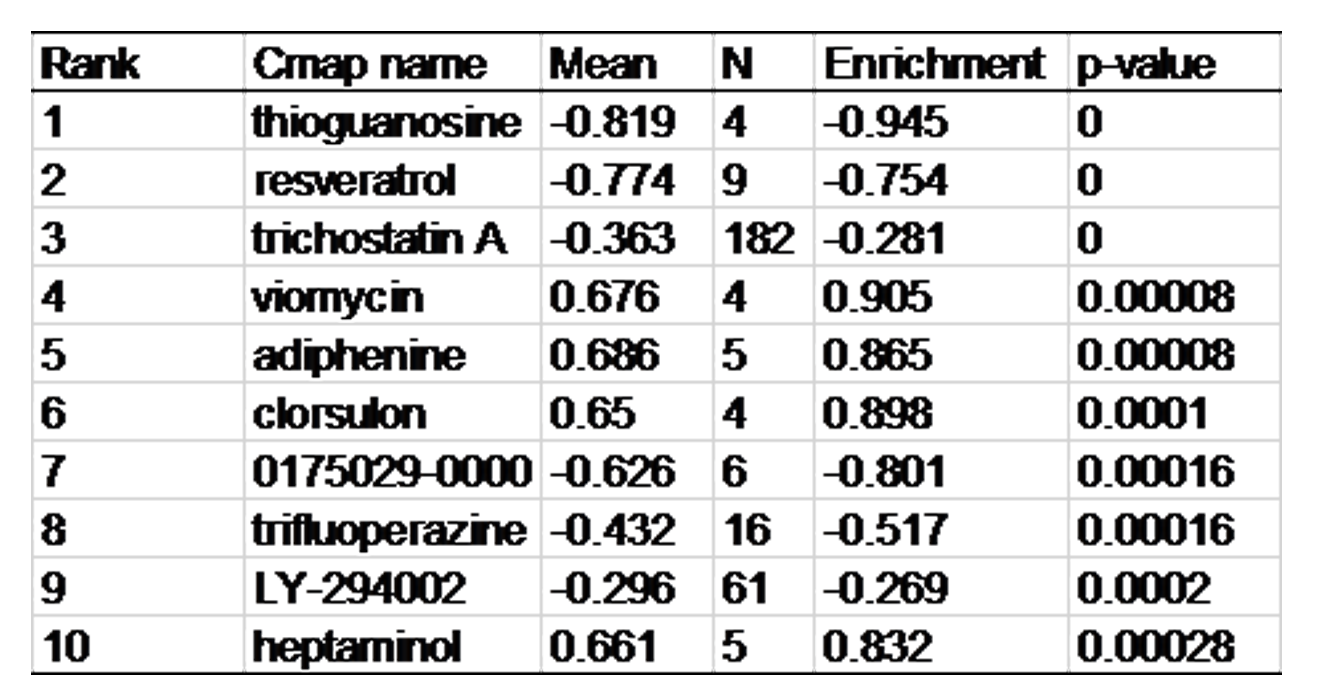

Supplement: Supplementary file 5 — Additional file 5: Figure S5. Results of CMap analysis. [file 12935_2020_1140_MOESM5_ESM.tiff]

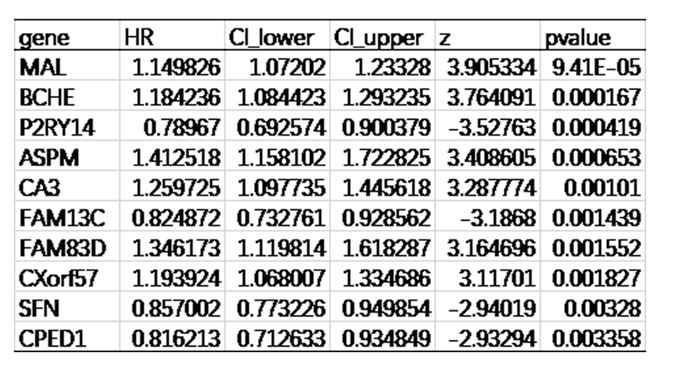

Supplement: Supplementary file 6 — Additional file 6: Figure S6. Univariate Cox proportional hazards regression analysis showed the top 10 EC-relative genes. [file 12935_2020_1140_MOESM6_ESM.tiff]

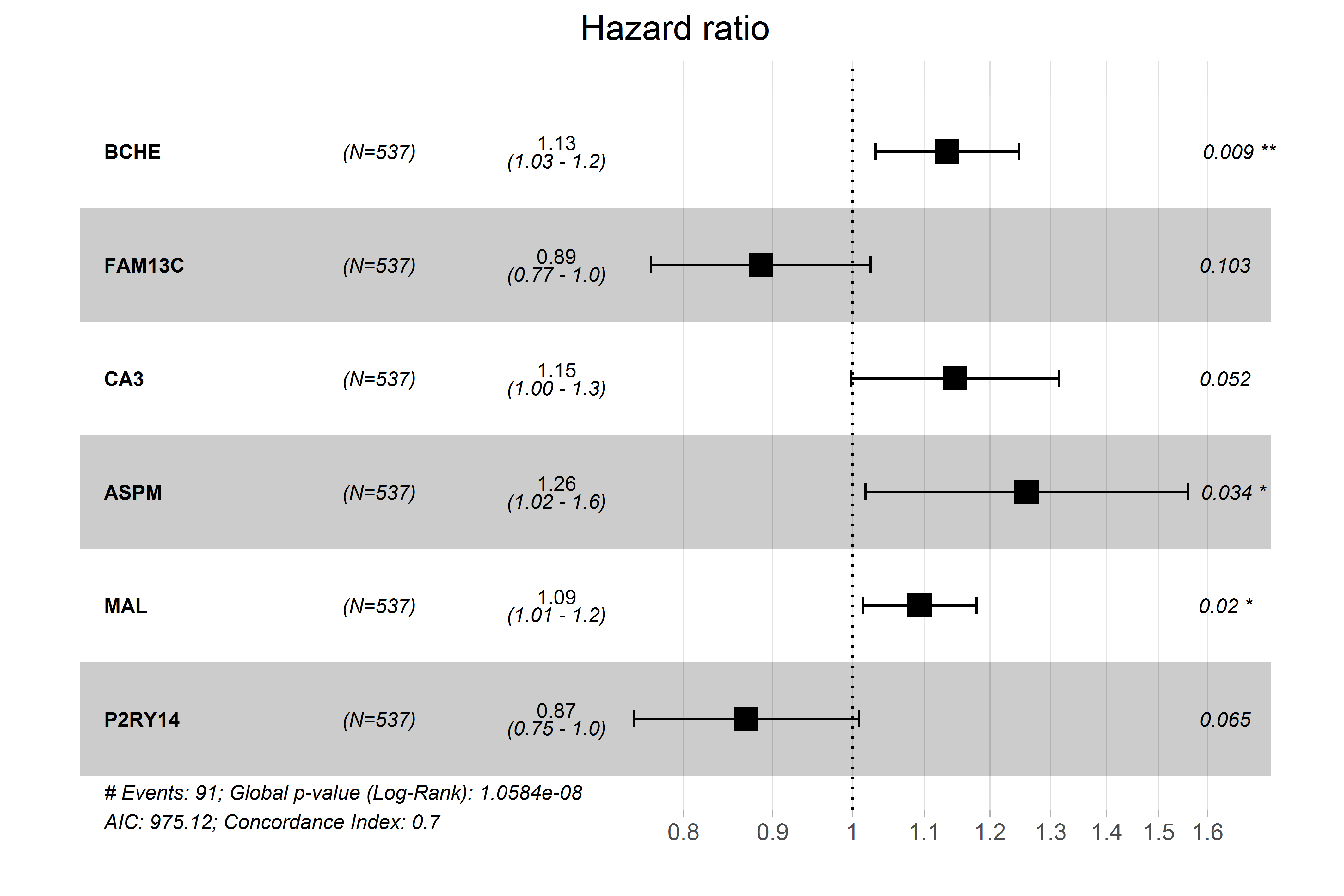

Supplement: Supplementary file 7 — Additional file 7: Figure S7. Multivariate Cox proportional hazards regression analysis further screened out 6 hub genes. [file 12935_2020_1140_MOESM7_ESM.tiff]

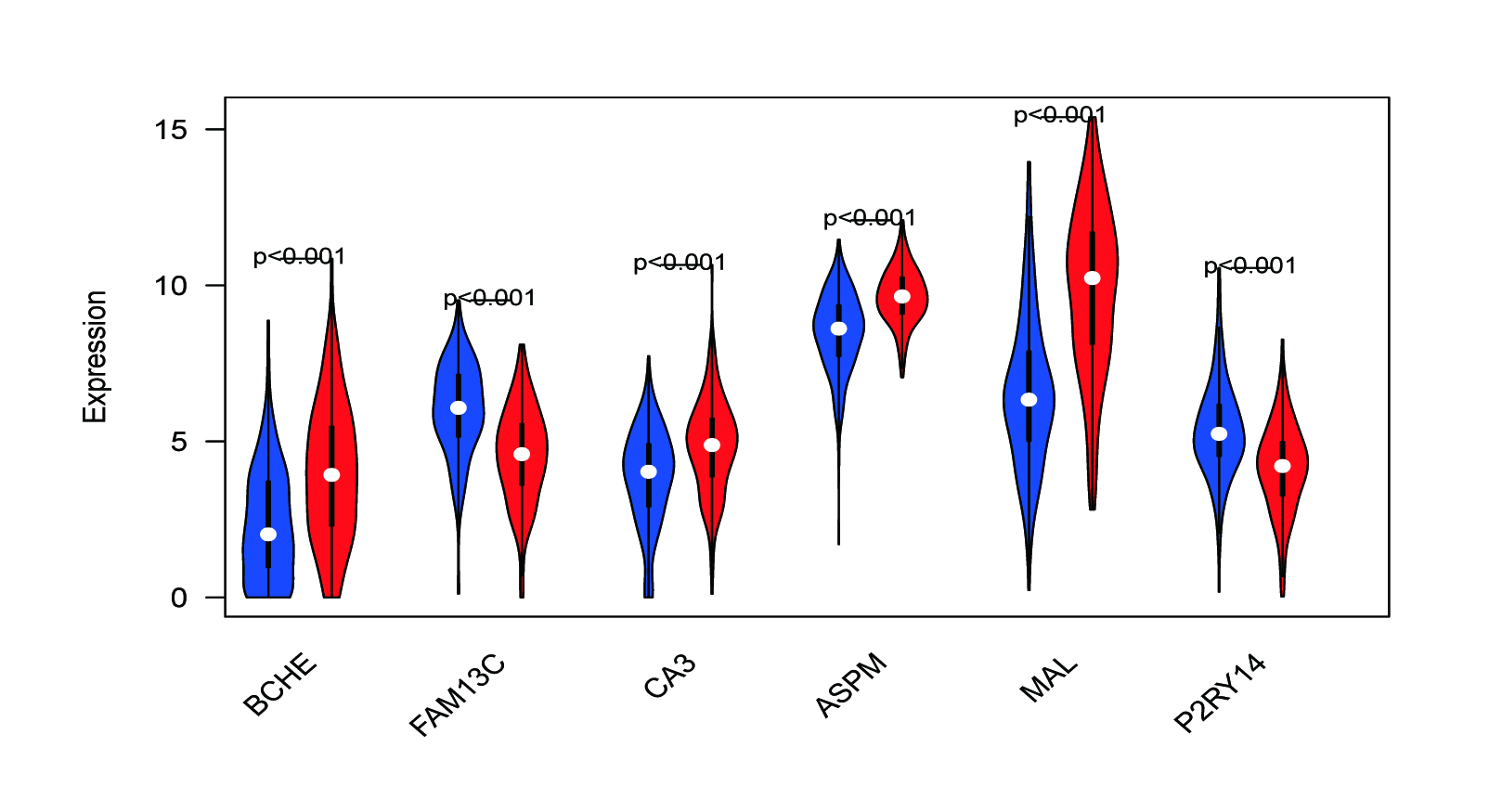

Supplement: Supplementary file 8 — Additional file 8: Figure S8. Expression of the six genes in low- and high-risk groups based on TCGA dataset .Red represents high-risk groups, blue represents low-risk groups. [file 12935_2020_1140_MOESM8_ESM.tif]

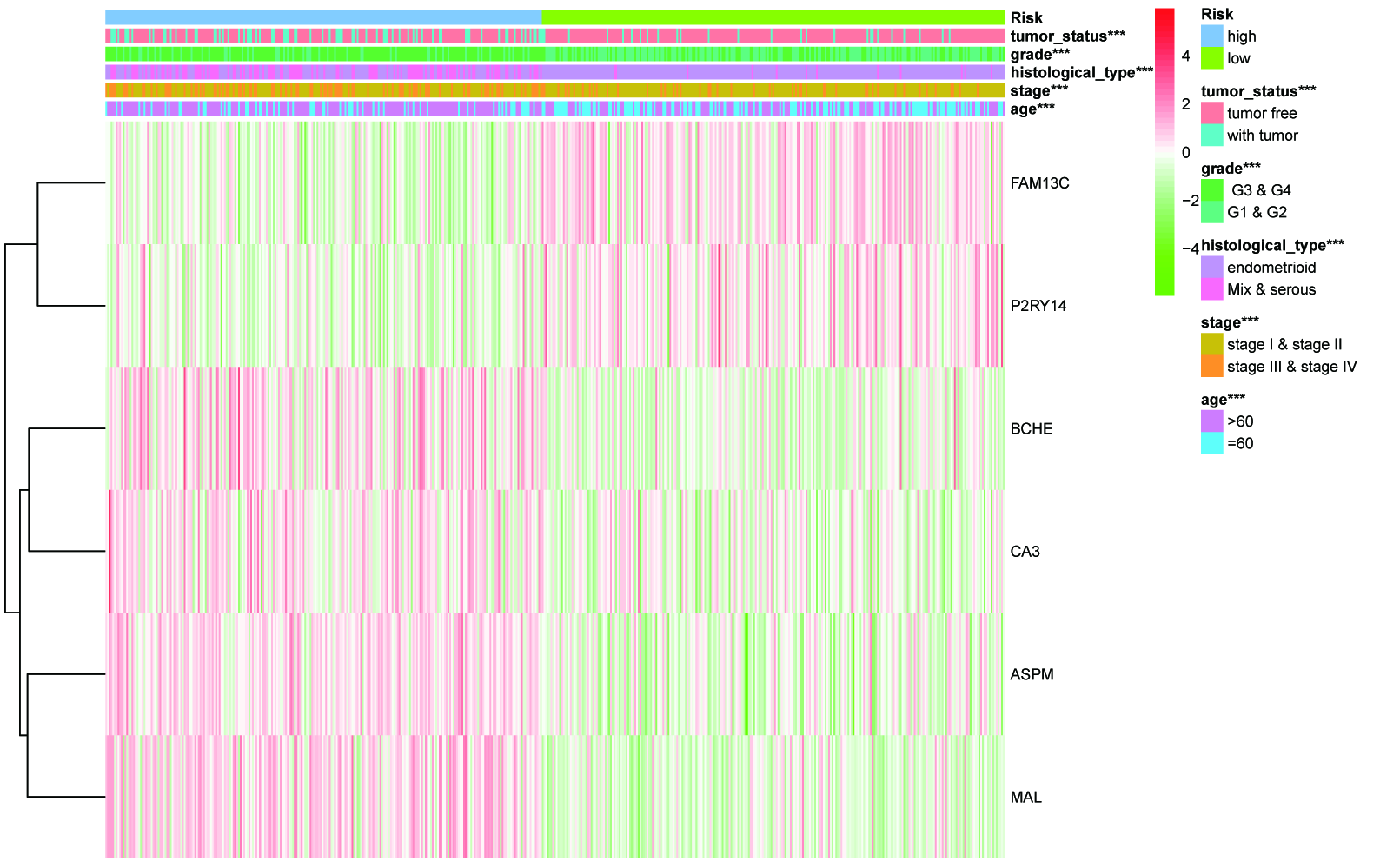

Supplement: Supplementary file 9 — Additional file 9: Figure S9. The heatmap of the six-gene expression levels between high- and low-risk groups in clinical information based on the TCGA dataset. [file 12935_2020_1140_MOESM9_ESM.tif]
